# Retrotransposition creates sloping shores: a graded influence of hypomethylated CpG islands on flanking CpG sites

**DOI:** 10.1101/gr.185132.114

**Published:** 2015-08

**Authors:** Fiorella C. Grandi, James M. Rosser, Simon J. Newkirk, Jun Yin, Xiaoling Jiang, Zhuo Xing, Leanne Whitmore, Sanum Bashir, Zoltán Ivics, Zsuzsanna Izsvák, Ping Ye, Y. Eugene Yu, Wenfeng An

**Affiliations:** 1 School of Molecular Biosciences, Washington State University, Pullman, Washington 99164, USA;; 2 Department of Pharmaceutical Sciences, South Dakota State University, Brookings, South Dakota 57007, USA;; 3 The Children's Guild Foundation Down Syndrome Research Program, Department of Cancer Genetics and Genetics Program, Roswell Park Cancer Institute, Buffalo, New York 14263, USA;; 4 Max Delbrück Center for Molecular Medicine, 13092 Berlin, Germany;; 5 Division of Medical Biotechnology, Paul Ehrlich Institute, 63225 Langen, Germany

## Abstract

Long interspersed elements (LINEs), through both self-mobilization and *trans*-mobilization of short interspersed elements and processed pseudogenes, have made an indelible impact on the structure and function of the human genome. One consequence is the creation of new CpG islands (CGIs). In fact, more than half of all CGIs in the genome are associated with repetitive DNA, three-quarters of which are derived from retrotransposons. However, little is known about the epigenetic impact of newly inserted CGIs. We utilized a transgenic LINE-1 mouse model and tracked DNA methylation dynamics of individual germline insertions during mouse development. The retrotransposed *GFP* marker sequence, a strong CGI, is hypomethylated in male germ cells but hypermethylated in somatic tissues, regardless of genomic location. The *GFP* marker is similarly methylated when delivered into the genome via the *Sleeping Beauty* DNA transposon, suggesting that the observed methylation pattern may be independent of the mode of insertion. Comparative analyses between insertion- and non-insertion-containing alleles further reveal a graded influence of the retrotransposed CGI on flanking CpG sites, a phenomenon that we described as “sloping shores.” Computational analyses of human and mouse methylomic data at single-base resolution confirm that sloping shores are universal for hypomethylated CGIs in sperm and somatic tissues. Additionally, the slope of a hypomethylated CGI can be affected by closely positioned CGI neighbors. Finally, by tracing sloping shore dynamics through embryonic and germ cell reprogramming, we found evidence of bookmarking, a mechanism that likely determines which CGIs will be eventually hyper- or hypomethylated.

Sequencing the human genome has revealed a wealth of information about the genetic code underpinning human development and disease. Although 1% of the human genome is protein coding, >46% is composed of transposable elements (TEs) (Lander et al. 2001; de Koning et al. 2011). Mammalian TEs are grouped into two major classes according to their mode of mobilization—the “copy and paste” retrotransposons and the “cut and paste” DNA transposons. Retrotransposons are further classified into three types—long interspersed elements (LINEs), short interspersed elements (SINEs), and long-terminal-repeat (LTR) retrotransposons. Both DNA transposons and LTR retrotransposons lost their mobility during primate radiation, whereas LINE-1s (L1s) and SINEs remain active in the human genome (Lander et al. 2001). In addition to replicating themselves, L1s are also responsible for the mobilization of SINEs and for the dispersal of two other classes of retrotransposed sequences (i.e., processed pseudogenes and transduction). Processed pseudogenes result from retrotransposition of spliced mRNAs (Esnault et al. 2000). Approximately 10% of human protein-coding genes have at least one processed pseudogene copy (Zhang et al. 2003), but the actual magnitude of processed pseudogenes may have been obscured due to 5′ truncation during retrotransposition. Indeed, a transcriptome-based search identified a large number of short pseudogenes that correspond to the 3′ UTR of cellular mRNAs (Terai et al. 2010). Three prime (3′) transduction occurs when the sequence downstream from an L1 is included as part of the L1 transcript and subsequently copied into the genome (Moran et al. 1999); it is found in ∼20% of L1 insertions (Goodier et al. 2000; Pickeral et al. 2000) and ∼10% of SVA insertions (Xing et al. 2006). A special case of 3′ transduction is orphan 3′ transduction, which lacks any retrotransposon sequence due to 5′ truncation. The magnitude of orphan 3′ transduction in the human genome can be substantial (Solyom et al. 2012).

The impact of retrotransposition on genomic architecture has been extensively documented (Hancks and Kazazian 2012; Huang et al. 2012). Data from the 1000 Genomes Project indicate that polymorphic germline insertions account for ∼25% of interindividual structural variations (Kidd et al. 2010; Lam et al. 2010). Any two individuals may differ by 600–2000 polymorphic insertions (Stewart et al. 2011). Importantly, retrotransposons continue to mutagenize human genomes. New germline insertions for *Alu*, L1, and SVA are estimated to occur one in every 20, 200, and 900 births, respectively (Xing et al. 2009), and are responsible for at least one in every 1000 spontaneous mutations in humans (Callinan and Batzer 2006). In addition to the insertion itself, retrotransposition also modifies the target site. New insertions are frequently accompanied by target site duplications (TSD) and/or deletions (Gilbert et al. 2002; Symer et al. 2002; Han et al. 2005). The target site is also subject to post-insertional modifications. One such process is nonallelic homologous recombination between existing copies (Han et al. 2008). Another process is the rapid shortening of the 3′ poly(A) tract, introducing somatic and germ cell mosaicism (Grandi and An 2013). The impact of retrotransposition on genomic structure and function is not limited to the germline genome. Recent genome-wide or targeted sequencing efforts indicate that somatic retrotransposition appears to be more rampant than in the germline, creating mosaic somatic genomes in cancer and neuronal cells (Babatz and Burns 2013; Reilly et al. 2013).

Significantly less is known about the impact of retrotransposition on the epigenome. DNA methylation is an epigenetic modification essential for normal mammalian development (Smith and Meissner 2013). In mammalian genomes, methylation occurs predominantly at the fifth carbon of a cytosine in the cytosine-phosphate-guanine (CpG) context. CpG dinucleotides are underrepresented in mammalian genomes due to spontaneous deamination of methylated cytosines (Bird 1980). Despite its overall deficiency, there are genomic regions where CpG frequency is closer to the expected (i.e., equivalent to the product of C and G frequencies). These regions are referred to as CpG islands (CGIs) (Bird et al. 1985). The human genome contains more than 50,000 CGIs, and approximately half of them reside in repetitive sequences, mainly TEs, including *Alus* and the promoter region of full-length L1s (Lander et al. 2001). The remaining CGIs are located in unique or low-copy sequences; among them, approximately half are associated with promoter regions, whereas the other half are within intra- or intergenic regions (Rollins et al. 2006). DNA methylation can serve as a regulatory switch for transcriptional initiation of genes with overlapping CGIs in their promoters (Deaton and Bird 2011). Similar roles in transcriptional regulation have been proposed for intragenic and intergenic CGIs, which may represent alternative promoters for coding or noncoding RNAs that regulate gene expression (Deaton and Bird 2011).

Retrotransposons have been proposed to act as epigenetic mediators of phenotypic variation based on early studies of specific LTR-retrotransposons (Whitelaw and Martin 2001). Consistent with this hypothesis, significant interindividual variability in DNA methylation has been observed for discrete *Alu* and L1 elements (Sandovici et al. 2005; Singer et al. 2012). In addition, monoallelically expressed genes are frequently flanked by high densities of evolutionarily recent L1s but low densities of SINEs (Greally 2002; Allen et al. 2003), implicating a role of differential epigenetic modification of retrotransposon subfamilies in controlling neighboring gene expression. Tissue-specific and subfamily-specific hypomethylation signatures have been identified in human embryonic and adult tissues, providing evidence that TEs may be responsible for wiring tissue–specific regulatory networks and may have acquired tissue-specific epigenetic regulation (Xie et al. 2013). Epigenetic regulation of non-LTR retrotransposons may also be important during disease processes. Cancer genomes are characterized by global hypomethylation and gene-specific hypermethylation (Baylin and Jones 2011). In tumor samples, L1s are variably hypomethylated, whereas hypermethylated genes have a lower frequency of L1s and SINEs near their transcription start sites, suggesting retrotransposons may modulate predisposition to DNA methylation in cancer (Estécio et al. 2010). In the male germline, proper remethylation of retrotransposons after genome-wide demethylation is crucial for spermatogenesis, and it is dependent on de novo DNA methyltransferases (DNMTs) and an intact piRNA pathway (Bourc'his and Bestor 2004; Aravin et al. 2008; Kuramochi-Miyagawa et al. 2008). Nevertheless, some members of younger retrotransposon families tend to evade piRNA-guided remethylation in male germ cells (Molaro et al. 2011, 2014).

Thus far, factors that dictate differential regulation of non-LTR retrotransposons and their influence on flanking sequences are poorly understood. In this study, we sought to address the impact of L1 retrotransposition on DNA methylation landscape by retrotransposing single-copy CGI sequence into the mouse genome and by analyzing methylomic data across tissues and developmental stages.

## Results

### Retrotransposed and transposed marker sequences are methylated in somatic but not germ cell lineages

**Figure 1.**
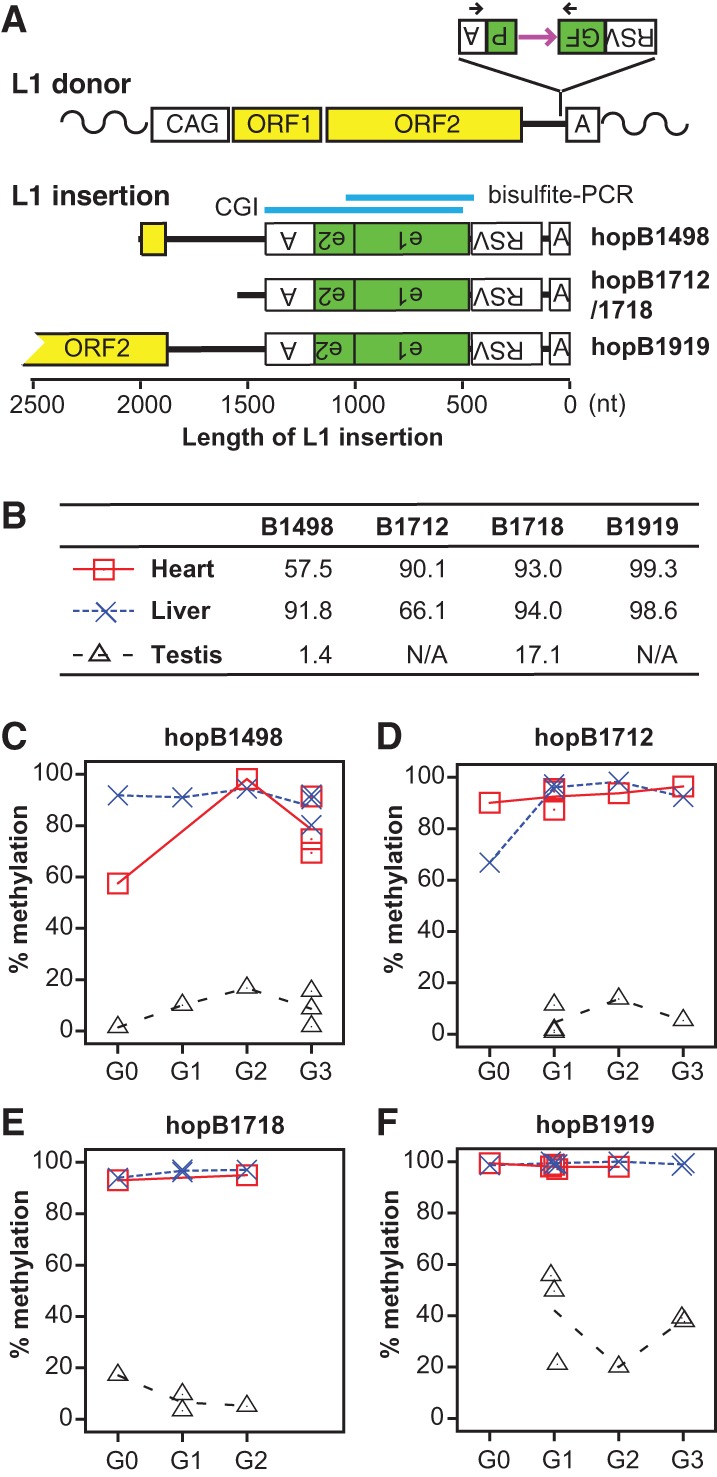
Integrating marker sequences into the mouse germline through L1 retrotransposition. (*A*) The donor L1 transgene *ORFeus* consists of a modified chicken beta-actin promoter (CAG), codon-optimized mouse L1 ORF1 and ORF2, a *GFP*-based retrotransposition indicator cassette in the 3′ UTR, and a polyadenylation signal (boxed letter A). The *GFP* cassette is placed in the antisense orientation relative to L1 transcription. The *GFP* reporter gene is flanked by Rous sarcoma virus (RSV) promoter and a polyadenylation signal but interrupted by a sense-oriented intron (purple horizontal arrow). Black arrows designate the location of genotyping primers. Retrotransposition creates a new L1 insertion, which is typically 5′ truncated, intronless, and trailed by a poly(A) DNA tract. Sequence structure of characterized L1 insertions is drawn to scale. All insertions are aligned at the 3′ end. For clarity, target site duplications and 3′ poly(A) tracts are omitted. Inverted letters indicate antisense orientation. For hopB1919, a near full-length insertion, only its 3′ portion is shown. Indicated *above* the insertions is the location of a 899-bp CGI predicted by newcpgseek as well as the region amplified by bisulfite PCR. (*B*) Methylation in *GFP* for all G0 animals. Animal IDs are indicated at the *top*. B1712 and B1718 are siblings with the same insertion. No testicular data are shown for B1919 and B1712 as both are female. (*C*–*F*) Contrasting methylation profiles between somatic tissues (heart and liver), and germ cells are maintained across multiple generations. Each point represents data from one individual animal. The connecting lines depict average methylation levels.

We previously developed *ORFeus*-based transgenic mouse models for L1 retrotransposition (An et al. 2006; Rosser and An 2010). These models feature a strong heterologous promoter and coding sequences from the synthetic L1 *ORFeus* (Fig. 1A). Unlike L1 transgenes with endogenous L1 promoters (Kano et al. 2009), the *ORFeus*-based models readily generate heritable insertions. The donor transgene was maintained in a hemizygous state by backcrossing to wild-type animals (Supplemental Fig. 1A). The progeny were PCR genotyped with an intron-flanking primer pair as previously described (Supplemental Table 1; An et al. 2006). The presence of an intronless band would indicate retrotransposition event(s). In this study, we were particularly interested in four animals (designated as G0 animals) that carried only the intronless band (Table 1; Fig. 1A). These animals were designated as G0 because they were the first in the lineage to segregate the insertion from the donor element. It is noteworthy that such insertions could either be an authentic germline retrotransposition event prior to meiosis (Ostertag et al. 2002) or have originated in the parent of G0 animals during embryogenesis (Kano et al. 2009) (e.g., hopB1712/1718; discussed below). Each insertion was propagated through the germline by backcrossing the G0 animal to wild-type animals (Supplemental Fig. 1A). Tissues from G0 and subsequent generations were collected and analyzed. The pedigree of each germline insertion was identified by the G0 animal ID (for example, the insertion carried by B1498 and progeny was termed hopB1498). Among the four G0 animals, B1712 and B1718 were littermates. Further experiments indicated that B1712 and B1718 had the same insertion located on Chromosome 2, which was inherited from their transgene-positive mother (Grandi et al. 2013).

**Table 1.**
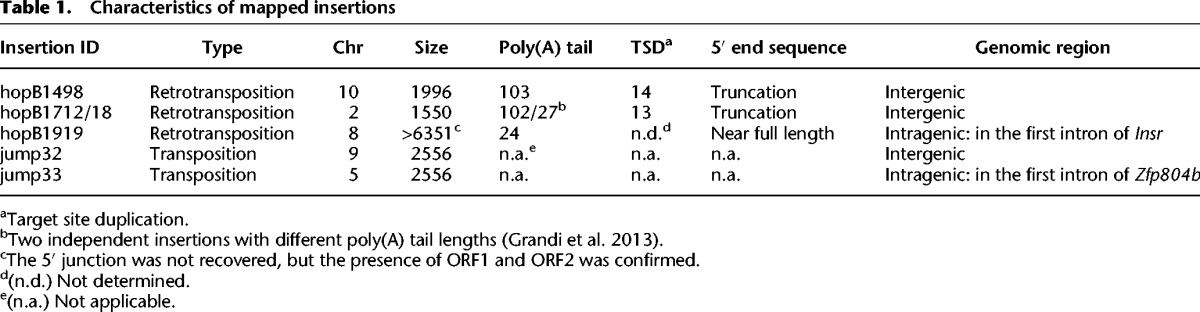
Characteristics of mapped insertions

Endogenous L1 insertions are highly methylated in somatic tissues (Rosser and An 2012). To examine the methylation status of each insertion launched from the *ORFeus* transgene, we performed bisulfite-sequencing analysis of the retrotransposed *GFP* sequence in heart and liver. The primer pair flanked the first *GFP* exon and specifically amplified the intronless insertion (Fig. 1A; Supplemental Fig. 1B). The *GFP* sequence was highly methylated in the heart and liver of G0 adult mice (Fig. 1B; Supplemental Fig. 1C–F). In contrast to somatic tissues, endogenous L1 insertions are known to undergo dynamic methylation changes in the germline (Rosser and An 2012). In the male germline, DNA methylation marks are erased from L1 promoters by embryonic day (E)13.5, restored through de novo DNA methylation by E17.5, and subsequently maintained throughout postnatal germ cell development (Hajkova et al. 2002; Lees-Murdock et al. 2003). To examine methylation dynamics in the germline, we first performed bisulfite sequencing with adult testes. Unexpectedly, the *GFP* sequence was significantly hypomethylated in the testis of G0 animals (Fig. 1B; Supplemental Fig. 1C–F). Further experiments with germ cells enriched from E14.5 and E18.5 embryos and testicular cells from postnatal day 6 (P6) and P20 animals suggested that the retrotransposed *GFP* sequence had been maintained in an unmethylated status after genome-wide demethylation in male germ cells (Supplemental Fig. 2). The lack of methylation at *GFP* marker sequence in postnatal germ cells contrasts with endogenous L1 5′ UTRs, which are highly methylated except among a subset of younger L1 families (Bourc'his and Bestor 2004; Aravin et al. 2008; Kuramochi-Miyagawa et al. 2008; Molaro et al. 2011, 2014). In this regard, the retrotransposed *GFP* acts as a surrogate for a 5′ UTR from a new L1 family. The observed somatic-high-and-germ-cell-low methylation pattern was transgenerationally maintained for all insertions characterized (Fig. 1C–F).

To examine whether the observed methylation patterns for the *GFP* reporter are specific to the process of retrotransposition, we mobilized the same *GFP* cassette by the *Sleeping Beauty* (*SB*) DNA transposon system. In this system, the *SB* transposase can mobilize any sequence flanked by two inverted terminal repeats (ITRs), which contain the transposase binding sites necessary for transposition (Ivics et al. 1997). We constructed an *SBGFP* transgene by placing the intronless *GFP* reporter between two ITRs and obtained a donor mouse line carrying approximately 40 copies of the *SBGFP* transgene in a tandem array (Supplemental Fig. 3A). To obtain single-copy germline *SBGFP* insertions, the donor mice were bred with H1t-*SB100X* transgenic animals, which express the hyperactive *SB* transposase (Mátés et al. 2009) specifically in pachytene spermatocytes (Supplemental Fig. 3B). As observed for the retrotransposed *GFP* marker sequence, the transposed single-copy *SBGFP* was hypermethylated in the liver but hypomethylated in the testis of G0 animals at two independent genomic locations (i.e., jump32 and jump33) (Table 1; Supplemental Fig. 3C). The differential methylation pattern was also maintained transgenerationally (Supplemental Fig. 3D,E).

### The retrotransposed CGI influences flanking DNA methylation patterns

**Figure 2.**
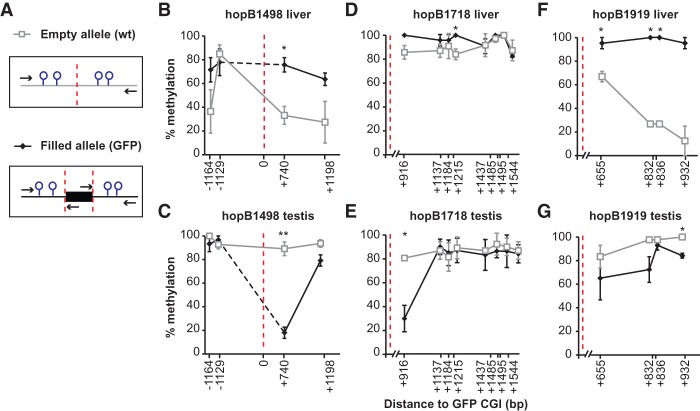
Comparison of DNA methylation at flanking CpGs between empty and filled alleles. (*A*) Schematic of the empty versus filled alleles and location of primers (arrows). For panels *B*–*G*, open gray rectangles indicate empty alleles, and closed black diamonds represent filled alleles. Error bars represent standard errors of the mean from three biological replicates. (*B*,*C*) HopB1498 flanking CpG methylation in adult liver and testis. Corresponding methylation dot plots are in Supplemental Fig. 4. The distance of each CpG to *GFP* CGI is marked on the *x*-axis (5′ and 3′ CpGs are indicated by − and + numbers, respectively). The insertion site is designated by a dashed red line. (*) *P* < 0.05; (**) *P* < 0.01. (*D*,*E*) HopB1718 flanking CpG methylation in adult liver and testis. No CpGs were interrogated upstream of the insertion site as it was CpG poor. (*F*,*G*) HopB1919 flanking CpG methylation in adult liver and testis.

The *GFP* marker sequence is highly CpG-rich. It contains a 899-bp-long CGI as predicted by the EMBOSS newcpgseek algorithm (Supplemental Fig. 1B; Rice et al. 2000). CGIs are often associated with transcription start sites and have an important role in gene regulation (Deaton and Bird 2011; Jones 2012). To determine the epigenetic consequence of a retrotransposed CGI on flanking genomic DNA sequences, we specifically amplified the insertion-containing “filled” allele and the corresponding “empty” allele (Fig. 2A). In this approach, the length of flanking regions analyzed was limited to ∼1 kb from the insertion site owing to bisulfite-induced DNA fragmentation. HopB1498 was located in a CpG-poor genomic region; accordingly, only two upstream and two downstream CpGs were interrogated (Supplemental Fig. 4A). In adult liver, the two 3′ flanking CpGs from the hopB1498 empty allele were moderately methylated (33.2% ± 7.6% and 27.4% ± 17.5% for CpGs at +740 and +1198, respectively) (Fig. 2B; Supplemental Fig. 4B). A greater than twofold increase was observed for both CpGs in the filled allele: Methylation at +740 CpG increased to 75.7% ± 6.1% (*P* = 0.013), whereas the more distant +1198 CpG increased to 63.6% ± 5.3% (*P* = 0.167). An opposite change in methylation was observed in the adult testis (Fig. 2C; Supplemental Fig. 4C). Both CpGs from the empty allele were highly methylated (89.2% ± 5.8% and 93.6% ± 3.2% for +740 and +1198 CpGs, respectively). In the filled allele, methylation at +740 CpG was significantly reduced (18.0% ± 4.8%; *P* = 0.002), but the more distant +1198 CpG had only a modest decrease (79.2% ± 4.8%; *P* = 0.301). No significant changes of DNA methylation were observed at the two upstream CpGs. These results suggest that the proximal CpG site at +740 in the filled allele has assumed the same methylation status as the retrotransposed CGI sequence.

Similar crosstalks were found in other insertions. HopB1718 insertion had eight flanking CpGs within ∼1 kb from its 3′ boundary (Supplemental Fig. 4D). In the liver, these CpGs were already methylated at high levels in the empty allele, and the additional increase in methylation in the filled allele was not statistically significant except at one CpG site (Fig. 2D). In testis, however, the closest CpG at +916 was significantly decreased in the filled allele when compared to the empty allele (*P* = 0.012) (Fig. 2E). HopB1919 insertion appeared to be near full-length, but only the 3′ junction was recovered. We were able to interrogate the methylation status of four CpGs in the 3′ flanking sequence (Supplemental Fig. 4E). In the liver, methylation was increased for all four CpGs in the filled allele when compared to the empty allele (Fig. 2F). In the testis, modest decreases in methylation were observed for three of four CpGs in the filled allele (Fig. 2G). Taken together, our data from all three insertions indicate that a positive correlation exists between DNA methylation status of the inserted CGI and the flanking sequence. In somatic tissues, the insertion was highly methylated, and there was an increase of methylation at the flanking CpGs. In the testis, the insertion was minimally methylated and there was a decrease of methylation at the flanking CpGs. Notably, the change of methylation tended to occur in CpGs proximal to the retrotransposed CGI.

### Hypomethylated CGIs affect methylation levels of surrounding CpGs in a graded manner

**Figure 3.**
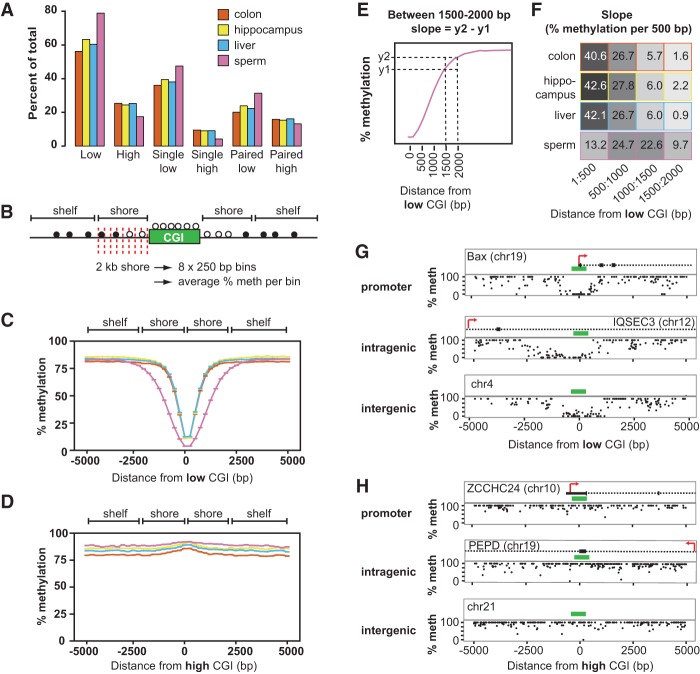
Hypomethylated CGIs create sloping CGI shores. (*A*) Percentage of 28,496 CGIs analyzed that fall into each classification (low, high, single low, single high, paired low, paired high) for each tissue analyzed in the repeat-masked human methylome. (*B*) Schematic of the process used to bin CpGs surrounding a CGI. Briefly, CpGs near each CGI were binned into 250-bp intervals, designated by the red lines, resulting in eight bins across the 2-kb shore. The average methylation for each bin was calculated and then averaged over all CGIs. (*C*) Average methylation of CpGs in 250-bp bins within 5 kb of single low CGIs (<20% methylation) in colon, hippocampus, liver, and sperm. Tissues are color coded as in *A*. Each point represents a bin average. Error bars represent standard errors of the mean (error bar heights may not be visible due to large sample sizes). Canonical shore and shelf regions are designated at the *top* of the graph. Intervals are marked by the starting coordinate, which indicates the distance of interval from the CGI; positive numbers are downstream; negative numbers are upstream of the CGI. (*D*) Average methylation within 5 kb of single high CGIs (>80% methylation). Tissues are color coded as in *A*. (*E*) Calculating the slope (change in % methylation over 500 bp). The average methylation levels at two 10-bp intervals y1 and y2 are used to calculate the slope at different ranges: 1–500, 500–1000, 1000–1500, 1500–2000. (*F*) Slopes for low CGIs in sperm and somatic tissues as in *C*. Heat map represents steepness of slope. Supplemental Figure 7A lists slopes for high CGI shores. (*G*,*H*) Representative sloping shores of low and high CGIs in three genomic contexts. Genes or genomic location for each example is given within the plot. Black boxes represent exons and 5′ UTRs, dashed lines represent introns, and green boxes designate the location of CGIs. Each dot represents one CpG. Plots do not include CpGs inside a CGI.

Based on our initial observation from *GFP* insertions, we sought to investigate if endogenous CGIs in the genome influence the methylation of the CpGs surrounding them. We analyzed methylomic data at single bp resolution in human and mouse cells and tissues (Supplemental Table 2; Molaro et al. 2011; Kobayashi et al. 2012, 2013; Hon et al. 2013; Ziller et al. 2013; Wang et al. 2014). CGIs were identified by newcpgseek in repeat-masked genomes, and islands with a length >200 bp were included in our initial analysis. Irrespective of the tissue type, >80% of all CGIs fell into one of the following two categories: either hypomethylated (i.e., with an overall level of methylation <20%) or hypermethylated (i.e., with an overall level of methylation >80%). For brevity, these CGIs were subsequently designated as low CGIs or high CGIs, respectively (Fig. 3A). In addition, to discern potential crosstalk between CGIs, we classified CGIs as either “single CGIs” if a CGI has no neighbors within 10 kb or “paired CGIs” if another CGI is located within 10 kb (Fig. 3A).

We first analyzed single CGIs in human sperm methylome (Molaro et al. 2011). CpG sites within a 5-kb distance of either side of the CGI were binned into 250-bp intervals, and the average methylation of each interval was calculated for each CGI (Fig. 3B). Analogous to our previous analysis on retrotransposed CGIs, we compared the behavior of low CGIs and high CGIs. For low CGIs, a graded effect on the nearby CpGs could be detected up to 2 kb away from either side of the CGI boundary (Fig. 3C). These regions were previously defined as CGI shores (Irizarry et al. 2009). Accordingly, we term this phenomenon “sloping shores” due to the graded influence of CGIs on nearby CpGs. No sloping was evident in regions located within 2–4 kb from either side of the CGI (known as CGI shelves) (Bibikova et al. 2011) as well as in the more distant “open sea” regions (Fig. 3C; Sandoval et al. 2011). In contrast, CpGs within the shore of a high CGI showed no significant change in methylation compared to the surrounding more distant CpGs (Fig. 3D). Similar results were obtained using 100-bp intervals (Supplemental Fig. 5A,B) as well as for the mouse sperm methylome, regardless of the strain analyzed (Supplemental Fig. 6A,B; Kobayashi et al. 2012; Wang et al. 2014).

We then determined the slope of CGI shores in human somatic tissues (Ziller et al. 2013). As in the sperm, the sloping shore phenomenon was only observed proximal to low CGIs in hippocampus, liver, and colon, whereas high CGI shores had no sloping (Fig. 3C,D). We observed that the average sloping shore was nearly identical among human hippocampus, liver, and colon, but they differed from the sloping shore in sperm (Fig. 3C). Similarly, mouse sperm and liver had differing sloping shores surrounding low CGIs, whereas high CGIs had no slope (Supplemental Fig. 6B,C; Kobayashi et al. 2012; Hon et al. 2013; Wang et al. 2014). To quantify the difference in sloping shore dynamics, we calculated the slope of the shore in four 500-bp intervals (Fig. 3E; Supplemental Fig. 5F,G). All three somatic low CGI shores had a steep slope in the first 500 bp. In contrast, the corresponding slope of the sperm low CGI shores was threefold shallower (Fig. 3F). At 500–1000 bp, the sperm and somatic shores rose at the same rate (Fig. 3F). At 1000–1500 bp, the somatic shores were nearing plateau methylation (Fig. 3C); this is accompanied by a fourfold decrease in the slope (Fig. 3F). In contrast, the sperm shores continued to rise with a slope similar to the previous interval but began to slow down as they approached plateau methylation at 1500–2000 bp (Fig. 3F). Beyond 1500 bp, somatic tissues had reached plateau methylation and showed minimal slope (Fig. 3F). As expected, high CGI shores had slopes of ∼0% (Supplemental Fig. 7A). These genome-wide findings were verified by inspecting individual CGIs. Although each island varied slightly from the genomic average, the rising shores were visible in low CGIs (Fig. 3G) but not at high CGIs (Fig. 3H) at promoters, intergenic, and intragenic regions.

CpG shores were discovered as hotbeds for cancer- and tissue-specific differentially methylated regions (cDMRs and tDMRs, respectively) (Irizarry et al. 2009). Although the average slope for low CGIs in somatic tissues was the same at the genomic level (Fig. 3C), by calculating the cumulative difference between two methylomes for individual shores, we were able to recover tDMRs and cDMRs that were obscured by the averaging approach (Supplemental Fig. 7C–F,G–I for individual examples). We also analyzed the methylome of a human embryonic stem cell line, HUES64, and its ectoderm, mesoderm, and endoderm derivatives (Gifford et al. 2013; Ziller et al. 2013); all cells displayed the same high-low shore slope dichotomy as the adult tissues (Supplemental Fig. 7B,F).

### The slope of a CGI is influenced by neighboring CGIs

**Figure 4.**
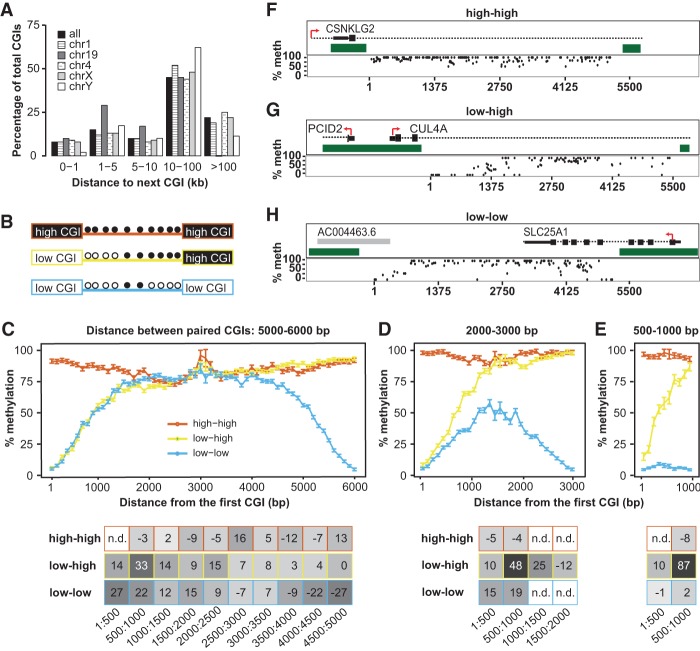
Neighboring CGIs influence shore slopes. (*A*) Percentage of CGIs that have a neighboring CGI within a given distance, either genome-wide or in selected chromosomes. (*B*) Schematic of possible pairs of CGIs: high-high, low-high, and low-low. Methylation status of CpGs between islands is indicated as filled (methylated) or open (unmethylated) circles. (*C*) Pairs of CGIs within 5000–6000 bp apart. Plot represents global average over 100-bp intervals. Error bars represent standard errors of the mean. The *x*-axis indicates distance of the CpG from the first CGI (on the *left*). Slope of the shore at various intervals is represented on the table *below* the plot. Points with n.d. did not have enough CpGs in the given interval to calculate a slope. Plots do not include CGI CpGs. (*D*) Pairs of CGIs within 2000–3000 bp apart. (*E*) Pairs of CGIs within 500–1000 bp apart. (*F*–*H*) Representative examples of CGI pairs in the genome for high-high (*F*), low-high (*G*), and low-low (*H*). Genomic context is pictured *above* each plot. Dark green blocks represent the CGIs. Red arrows indicate the direction of transcription. Black boxes and dashed lines denote annotated genes. Gray boxes denote annotated transcripts. Each dot represents one CpG. Plots do not include CpGs inside a CGI. The *x*-axis indicates distance from the CGI on the *left*.

Heretofore, our analysis has focused on CGIs in isolation from each other. However, one-third of CGIs in the repeat-masked human genome have a CGI neighbor <10 kb away (Fig. 4A). Due to the ability of low CGIs to influence methylation of flanking CpGs in their shores, we reasoned that one CGI might alter the slope of its neighboring CGI. Fortuitously, the hopB1919 insertion contained five >200-bp CGIs spanning a 5-kb region in the ORF1 and ORF2 sequence (Supplemental Fig. 8A,B), providing an opportunity to study retrotransposed CGIs in pairs. In the heart and liver, all the CGIs and the surrounding CpGs surveyed were hypermethylated (>80%) (Supplemental Fig. 8C,D). In contrast, in the testis, the three internal CGIs were hypermethylated (>80%), but the two outer CGIs were relatively hypomethylated (∼40%) (Supplemental Fig. 8E). Interestingly, the CpGs between the hyper- and hypomethylated CGIs displayed intermediate levels of methylation. As the distances between the hyper- and hypomethylated CGIs were shorter than standard CGI shores (2 kb), it provided evidence that the presence of high CGIs in close proximity to low CGIs counteracted the influence of low CGIs (Supplemental Fig. 8E).

To extend our analysis to the genome, we analyzed CGI pairs in the human sperm methylome (Molaro et al. 2011). Paired CGI were defined as any two CGIs within 10 kb of one another and classified according to their methylation status (e.g., low-low, low-high, and high-high) (Fig. 4B,F–H for individual examples). We incrementally decreased the distance between the two CGIs and interrogated the methylation status of the intervening CpGs. When two CGIs were separated by 5000–6000 bp or more, the sloping shore dynamics mirrored those of single CGIs (Fig. 4C). For low-low pairs, both CGIs had graded slopes outward from the island that reach a methylation plateau at ∼2000 bp away from the respective CGI. For high-high pairs, intervening CpGs were found at the background methylation level. For low-high pairs, graded slopes were present near the low CGI, with the same rate as their low-low counterparts, but the adjoining high CGI had a slope of nearly zero. However, as the distance between paired CGIs decreased, crosstalk between low-low and low-high pairs became evident (Supplemental Fig. 9). At 2000–3000 bp away, CpGs between two low CGIs experienced a depression in methylation, compared to CpGs within a similar distance away from single CGIs (33% and 50% methylation at 1000 and 1500 bp away in such low-low pairs compared to 56% and 64% methylation for single CGIs, respectively) (Fig. 4D). Unlike low-low pairs separated by 5000–6000 bp, those at a 2000–3000 bp distance never reached the plateau methylation level. Likewise, CpGs between low-low pairs separated by 500–1000 bp had a 28-fold reduction in methylation compared to CpGs at the same distance away from single CGIs, and methylation levels never rose above 15% (Fig. 4E). These observations suggested that low-low pairs positively feed back on the presence of a neighbor, decreasing surrounding CpG methylation more than would be expected. The effect of a neighboring high CGI was interrogated using the low-high pairs. As in the low-low pairs, a crosstalk effect was observed as the islands moved closer together. At 2000–3000 bp apart, the slope of the low CGI shore became steeper, resulting in plateau methylation being reached earlier (Fig. 4D). The steepening of the slope further intensified when low-high CGIs became 500–1000 bp apart (Fig. 4E). These results suggest that the presence of a high CGI acts to counteract the effects of the low CGI. In other words, the surrounding CpGs are less likely to be demethylated, despite being situated in the shore of a low CGI. Similar crosstalk effects were observed in the liver methylome (Supplemental Fig. 10; Ziller et al. 2013).

### Sloping shore dynamics distinguish future-low CGIs from future-high CGIs during two episodes of DNA methylation reprogramming

**Figure 5.**
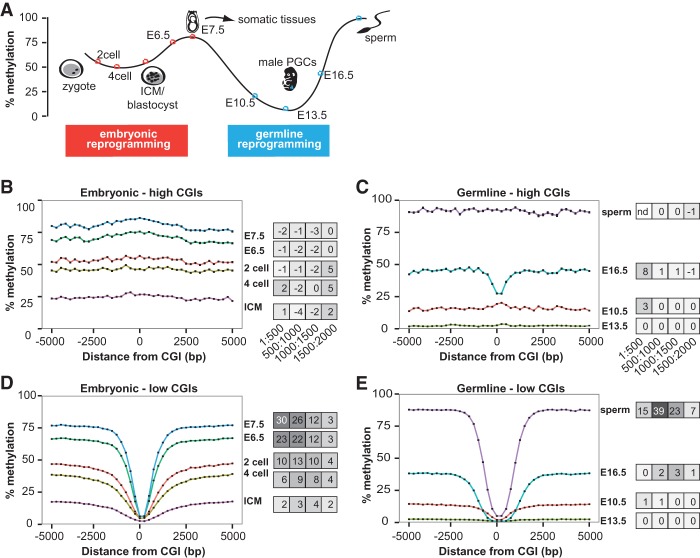
Sloping shore dynamics distinguish future-low and future-high CGIs during reprogramming. (*A*) Schematic of DNA methylation reprogramming through embryogenesis and germ cell development in the mouse. Colored dots represent time points that were analyzed in this study—red for embryonic and light blue for germ cells. (*B*) Methylation dynamics of future-high CGIs during embryonic development. High and low CGIs were defined based on their methylation status at E7.5 and traced through development. The developmental time point is labeled to the *right* of the line. Error bars represent standard errors of the mean (error bar heights may not be visible due to large sample sizes). Slopes at different intervals are depicted in a heat map to the *right* of the plot. (*C*) Methylation dynamics of future-high CGIs during germ cell reprogramming. High and low CGIs were defined based on their methylation status in sperm. (*D*) Methylation dynamics of future-low CGIs during embryonic reprogramming. (*E*) Methylation dynamics of future-low CGIs during germ cell reprogramming.

DNA methylation undergoes genome-wide reprogramming during both early embryogenesis and germ cell specification (Lee et al. 2014). We reasoned that important insights into the genesis of high and low CGIs could be gained by following the dynamics of the sloping shores through these reprogramming events (Fig. 5A). In early embryonic reprogramming, we analyzed mouse methylomes from two-cell, four-cell, inner cell mass (ICM), E6.5, and E7.5 embryos (Wang et al. 2014). CGIs were classified as future-high or future-low based on the eventual E7.5 methylome. For reprogramming in germ cells, we analyzed E10.5, E13.5, and E16.5 mouse germ cells and sperm (Kobayashi et al. 2012, 2013). Future-high and future-low CGIs were designated based on the sperm methylome. Mapping methylomic data to CGIs recapitulated known CGI reprogramming dynamics in early embryos (Smith et al. 2012; Wang et al. 2014) and during germ cell reprogramming (Seisenberger et al. 2012; Kobayashi et al. 2013). Namely, during embryonic reprogramming, future-low CGIs in the E7.5 methylome remained hypomethylated from two-cell stage forward (Supplemental Fig. 11A). Likewise, future-low CGIs in the sperm remained hypomethylated from E10.5 forward (Supplemental Fig. 11B). Future-high embryonic CGIs were intermediately methylated at the two-cell stage, dropped to 22% at the ICM stage, and then increased to their final hypermethylated state (Supplemental Fig. 11A). Likewise, future-high CGIs in the sperm began hypomethylated at 24% at E10.5, decreased to 4% at E13.5, remethylated to 23% at E16.5, and were fully methylated in the sperm (Supplemental Fig. 11B).

Remarkably, despite being similarly hypomethylated in either ICM or E13.5 germ cells, future-low and future-high CGIs were distinguished at these early time points by the difference in the slopes of their shores. Like high CGIs in adult somatic tissues, both embryonic and germ cell future-high CGIs had no visible sloping shores and remained at the genomic background methylation level consistent with the developmental point (Fig. 5B,C). For example, future-high CGI shores showed uniform methylation at 25% in the ICM, and then rose to 70% at E6.5 (Fig. 5B). In contrast, the future-low CGIs had sloping shores at all developmental time points, and the slope of the shores fluctuated as the genome was first demethylated and then remethylated (Fig. 5D,E). In the two-cell stage, at 1–500 bp away, the slope was 10% per 500 bp (Fig. 5D). As the genomic methylation level decreased at the four-cell and ICM stages, the slope also decreased to 6% and 2%, respectively (Fig. 5D). As the genomic level of methylation began to rise, so did the slope. Compared to ICM, the slope increased by 12-fold to 23% per 500 bp at E6.5 and by 15-fold to 30% at E7.5 (Fig. 5D). Likewise, a similar progression of slope dynamics was observed in the germ cell reprogramming in the 500–1000 bp interval, where the characteristic rise in the sperm shores occurred (see Fig. 3G). At E10.5, as the male germ line genome began to be demethylated, the slope was 1%, a 32-fold drop from the slope at the E7.5 methylome, and continued to drop to 0.1% at E13.5 (Fig. 5E). At E16.5, when de novo methylation had commenced, the slope gradually rose to 2% and finally to 39% at the sperm, a 19-fold increase (Fig. 5E).

### Retrotransposons are a major source of sloping shores in the human genome

**Figure 6.**
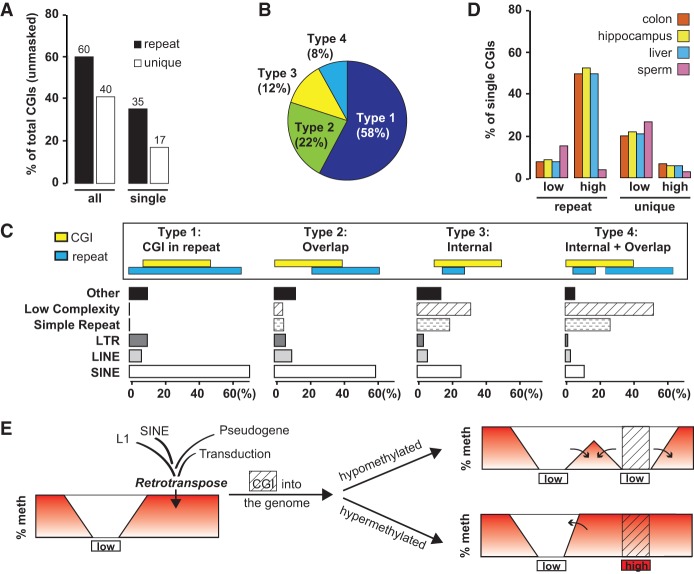
Retrotransposons create CpG islands and sloping shores at the genomic scale. (*A*) Abundance of repeat CGIs and unique CGIs in the unmasked human genome. (*B*) Proportion of the four different types of repeat-associated CGIs. The schematic in *C* illustrates the relationship between a CGI and a repeat(s) in each type. (*C*) Contribution of individual repeat categories to each type of repeat CGI. (*D*) Comparison of repeat and unique CGIs according to the methylation status in sperm and somatic tissues. (*E*) A model for changing the DNA methylation landscape by retrotransposed CGIs. See text for details.

So far, our analyses of sloping shores have focused on CGIs in the nonrepeat portion of the human and mouse genomes. To address the genome-wide contribution of retrotransposons in the formation of CpG islands and shores, we predicted CGIs from the entire (i.e., unmasked) human genome and categorized them into “unique CGIs” or repeat-associated CGIs (“repeat CGIs,” in short). Repeat CGIs make up ∼60% of all islands in the unmasked human genome, highlighting the importance of repeat elements in shaping the DNA methylation landscape (Fig. 6A). To understand the relative contribution of different classes of repeats to the CGI landscape, we annotated the repeat CGIs into four categories (Fig. 6B,C). In type 1, a CGI is completely contained within a RepeatMasker annotated genomic repeat. In type 2, a CGI partially overlaps with a repeat. The majority of the repeats found in type 1 and type 2 CGIs are SINEs (accounting for 71% and 60% of the CGIs in each category) (Fig. 6C). In type 3, a CGI has an internal repeat. In type 4, a CGI not only contains a repeat but also partially overlaps with another repeat (i.e., a mixed type 2 and type 3). Simple repeats and low complexity repeats together contribute to the majority of type 3 and type 4 CGIs (50% and 79% of the CGIs, respectively) (Fig. 6C). Although SINEs, LINEs, and LTR retrotransposons occupy 13%, 20%, and 8% of the human genome (Lander et al. 2001), our analysis shows that they are involved in 58%, 7%, and 8% of repeat CGIs, respectively (Fig. 6B,C). This discrepancy highlights the difference in CpG density among retrotransposon families: *Alus* are GC-rich over the entire length, whereas L1s are GC-poor except in the 5′ UTR of full-length L1s, which represent only a minor fraction of genomic L1 copies. To compare whether repeat CGIs possess similar shore slopes as unique CGIs, we also identified single-repeat and single-unique CGIs in the unmasked genome (i.e., no other CGIs within 10 kb) (Fig. 6A). Similar to our previous analysis of single CGIs in the masked human genome (Fig. 3A), the single-unique CGIs in the unmasked genome were predominantly hypomethylated in both somatic and germline tissues (Fig. 6D). This observation is not surprising because these two sets of CGIs largely overlap with each other. In contrast, single-repeat CGIs were generally hypermethylated in somatic tissues. However, in sperm, only a small proportion of the single-repeat CGIs were hypermethylated, and most single-repeat CGIs had intermediate levels of methylation (i.e., between 20% and 80%) (Fig. 6D). For each tissue, the slopes were nearly indistinguishable between unique CGIs and repeat CGIs (Supplemental Fig. 12), suggesting that the sloping shore phenomenon is an intrinsic property of CGIs regardless of the origin.

## Discussion

This study sought to determine the epigenetic impact of L1 retrotransposition at the target site. A *GFP*-based marker sequence, which has the characteristics of a strong CGI (Gardiner-Garden and Frommer 1987; Illingworth and Bird 2009), was retrotransposed by an engineered L1 retrotransposon to discreet locations in the mouse germline genome. Differential methylation in the *GFP* CGI was observed in mice carrying these germline insertions. The CGI was consistently hypermethylated in somatic cells but hypomethylated in male germ cells. This pattern of methylation was stably maintained through multiple generations and appeared to be independent of the genomic locations analyzed. The same pattern of methylation was observed when an identical *GFP* marker sequence was introduced into the mouse germline genome by a synthetic *SB* DNA transposon. These results suggest that the differential methylation pattern in the *GFP* sequence may be independent of the mode of insertion (i.e., the copy-and-paste retrotransposition versus the cut-and-paste transposition). The dynamics of *GFP* methylation was tracked during spermatogenesis at multiple time points. The results are consistent with a timeline in which the *GFP* CGI remains unmethylated in developing germ cells but becomes hypermethylated during early embryogenesis in the soma. Previously, two other studies reported the epigenetic silencing of retrotransposed *GFP*-based reporters in cultured cells (Muotri et al. 2005; Garcia-Perez et al. 2010). In both studies, the levels of methylation were inferred from the effect of treatment with a demethylating agent. To gain insight into DNA methylation of somatically retrotransposed *GFP* CGI, we performed bisulfite sequencing in the heart and liver of donor-positive adults and E14.5 embryos (Supplemental Fig. 13). In contrast to germline *GFP* insertions, the somatically retrotransposed *GFP* was hypomethylated in the heart and liver at both adult and E14.5 time points. Because the donor L1 transgene was always present, we could not pinpoint the timing of these somatic retrotransposition events. Nevertheless, these data hint at the possibility that the differentiating and/or differentiated somatic cells are incapable of methylating the newly retrotransposed *GFP* marker sequence.

By analyzing individual germline insertions and multiple published methylomes, we discovered “sloping shores”, i.e., a graded influence of hypomethylated CGIs on nearby CpGs within 2 kb from either side of the CGI. No sloping is evident in the more distant CGI shelves and open seas. CpG island shores were first reported in the context of cancer- and tissue-specific methylation (Irizarry et al. 2009). Prior to this landmark report, it had often been assumed that most DNA methylation changes in cancer would occur in promoter-associated CGIs. Instead, methylation arrays provided an unexpected view of the methylation landscape in cancer: Most methylation alterations in colon cancer occur in CGI shores rather than promoters or CGIs (Irizarry et al. 2009). These cDMRs distinguish normal tissues from colon, lung, breast, thyroid, and Wilms’ tumors (Hansen et al. 2011). Importantly, an inverse correlation between differential gene expression and differential DNA methylation at CGI shores has been observed in normal tissues, in cancers, in reprogrammed cells, and during lineage-specific differentiation (Doi et al. 2009; Irizarry et al. 2009; Ji et al. 2010). Mechanistically, CGI shores may serve as sites of alternative transcription and enhancer binding (Irizarry et al. 2009). Methylation changes in CGI shores may perturb the normal sharply defined island/shore boundary, underlying altered gene expression in cancer (Hansen et al. 2011). In contrast to hypomethylated CGIs, globally, we detected no sloping shores for hypermethylated CGIs when they are situated 10 kb away from other CGIs. However, two neighboring CGIs exert influence on one another if they are located within ∼3000 bp. For a hypomethylated CGI, the slope of its shore is steepened by a hypermethylated CGI neighbor, but lessened by a hypomethylated CGI neighbor. This crosstalk between nearby CGIs suggests that a CGI should not be studied in isolation because methylation changes in one CGI may affect other CGIs in its vicinity. It is noteworthy that the sloping shore phenomenon is not limited to CGIs that are >200 bp in length. Hypomethylated CGIs of 100–200 bp long also demonstrate similar sloping shores (Supplemental Fig. 5E), suggesting that sloping shores are length-independent. Thus, shorter CGIs should also be considered when monitoring methylation in CGI shores.

By examining sloping shore dynamics during development, we found that CGIs destined to be hypomethylated appear to have been bookmarked prior to the de novo methylation phase for both embryonic and germ cell reprogramming. Although these CGIs remain minimally methylated for the entire duration of the respective reprogramming process, the slope of the corresponding shores changes dynamically (first flattens and then deepens) as the genome (represented by regions outside the shores) experiences tidally falling and rising of DNA methylation levels. The putative bookmarking may be mediated by *trans*-acting factors, such as DNA-binding proteins and/or specific histone modifications, which may ultimately be determined by the *cis* DNA sequence. Transcription factors (TFs) are prime candidates (Lienert et al. 2011). The high GC content in CGIs increases the likelihood of containing TF binding sites, which are on average GC-rich (Deaton and Bird 2011). TF binding may protect the underlying CGIs from being methylated. A well-known example is SP1, which binds to unmethylated binding motifs and prevents flanking CpGs from methylation (Brandeis et al. 1994; Macleod et al. 1994). Other DNA binding motifs may also be involved (Straussman et al. 2009). Additional CGI interpreters include CxxC domain-containing proteins, such as CFP1, KMT2A, KDM2A, and KDM2B, all of which preferentially bind to unmethylated CpGs. Notably, they are all histone-modifying enzymes and serve important roles in maintaining local chromatin architecture (Blackledge et al. 2010; Cierpicki et al. 2010; Thomson et al. 2010; Farcas et al. 2012). Thus, it is possible that a CGI's unique chromatin structure may play a role in shielding it from the methylation machinery. Under this model, the protective factors, regardless of their nature, are not perfectly confined within the CGIs themselves as reflected by the graded influence of hypomethylated CGIs on surrounding shores. Proximal CpGs (within 1–500 bp from a CGI) are most likely to be protected by these marks. CpGs that are further away (500–1500 bp) are less likely to be protected, resulting in intermediate methylation levels. CpGs that are distally located in the CGI shores (1500–2000 bp away) are rarely, if ever, protected from methylation and consequently assume the high, default level of methylation in that tissue (i.e., plateau). As such, the observed gradation in sloping shores may be considered as the probability that a CpG site near a CGI can be accessed by DNMTs.

In contrast to hypomethylated CGIs, the dynamics of DNA methylation for CGIs destined to be hypermethylated are distinctly different. Methylation levels in these CGIs are seen to wane and wax along with the rest of the genome during the reprogramming process. For most of the time points interrogated, there is no discernable slope at the CGI shores. The only exception is found in E16.5 male germ cells, in which the remethylation of CpGs within 500 bp from the boundary of CGIs is delayed, forming a shallow valley in an otherwise methylated plateau (Fig. 5C). The significance of this delay is unknown. It may be related to the intrinsic kinetics of de novo methylation. It is possible that the increased density of CpGs in CpG islands and shores requires longer time to be methylated to the same level as compared to the average genomic regions. Nevertheless, these CGIs and the corresponding shores become fully methylated in the sperm. The contrasting methylation dynamics for hypo- and hypermethylated CGIs and their respective slopes beg an important question: How are these two types of CGIs differentiated by the DNA methylation machinery? If hypomethylated CGIs are bookmarked during the de novo methylation phase, as discussed above, it is necessary for this bookmarking system to spare those hypermethylated CGIs, which will then be treated as any other unprotected genomic regions and remethylated indiscriminatingly, in agreement with the notion that methylation is the default state of genomic DNA (Edwards et al. 2010). Genome-wide profiling of candidate transcription factors and histone markers during embryogenesis or germ cell development would help elucidate if such factors are acting to bookmark islands and other genomic features.

Until recently, TEs had been excluded from genome-wide CGI analyses because they were thought to exert no influence on gene expression. Accordingly, various strategies had been adopted to remove retrotransposons from the identified CGI library, such as by focusing only on the repeat-masked genome or by revising the selection criteria to exclude *Alus* (Takai and Jones 2002). Since then, studies conducted at both gene and genome levels have uncovered many TE insertions that have been co-opted for critical roles in gene regulation (Rebollo et al. 2012; de Souza et al. 2013). Indeed, TEs constitute an important source for the evolution of new CGIs. For example, approximately 1000 copies of SVA retrotransposons have been inserted into human genomes since the divergence from chimpanzees (Mikkelsen et al. 2005). Each copy of SVA contains a CGI that fulfills the more stringent CGI criteria by Takai and Jones (2002). Importantly, these human-specific SVA-derived CGIs are enriched with so-called “CpG beacons,” distinct genomic features that are associated with CGI evolution, human trait, and disease (Bell et al. 2012). L1 retrotransposition also creates CGIs in the form of processed pseudogenes. Many pseudogenes are imprinted, manifesting parent-of-origin specific methylation in the overlapping CGIs. In several cases, the imprinted intronic pseudogenes are also responsible for the imprinting of the corresponding genes that contain them (Cowley and Oakey 2010; Kanber et al. 2013). In our study, the *GFP* CGI retrotransposed into Chromosome 2 was not imprinted since no change in methylation patterns was observed when it was transmitted through either the female (B1712) or male (B1718) germline. This result is not unexpected because it has been suggested that the epigenetic fate of the retrotransposed DNA depends on its sequence and selective forces at the integration site (Kanber et al. 2013).

The present study provides a snapshot of the host response to a newly introduced CGI and suggests an important pathway by which L1-mediated retrotransposition can influence the epigenetic landscape of a mammalian genome. New CGIs can be part of an L1, a SINE, a processed pseudogene, or 3′ transduction of the downstream sequence by an L1. Not only can these CGIs cause epigenetic variations as tDMRs, alterations in DNA methylation extend beyond the CGI boundary into flanking CpGs, which are now part of the newly formed shores. Depending on the methylation status of the new CGI, the flanking CpGs in the newly created shores may be influenced to become hyper- or hypomethylated (Fig. 6E). This influence is more pronounced for hypomethylated CGIs but hypermethylated CGIs can also alter the shore slopes of neighboring hypomethylated CGIs through crosstalk. In this regard, it is noteworthy that members of younger retrotransposon families tend to evade piRNA-guided remethylation in male germ cells (Molaro et al. 2011, 2014). Furthermore, our observation that all somatically acquired *GFP* CGIs are unmethylated in somatic tissues (Supplemental Fig. 13) has important implications, especially in the context of recent findings that somatic retrotransposition appears to be more rampant than in the germline (Babatz and Burns 2013; Reilly et al. 2013). Because CpG methylation is associated with the level of transcription and the chromatin state (Deaton and Bird 2011; Jones 2012), these islands would introduce subtle changes to the epigenome and could over time build up an epigenetically plastic genome. Furthermore, the epigenetic impact of retrotransposition is not limited to the formation of new CGIs and the corresponding sloping shores per se. In fact, not all retrotransposition events create new CGIs. Examples include many 5′ truncated L1s that lack the 5′ UTR CGI. These L1 insertions can, however, alter the DNA methylation landscape by disrupting existing CpG islands and shores. Therefore, polymorphic L1-mediated insertions may explain some common quantitative traits through associated genetic and epigenetic variations. Although the mechanisms and “rules” determining which CGIs are methylated are still unclear, this study illustrates the utility of L1 mobilization to answer these questions. Future experiments with 5′ UTR sequences from different L1 subfamilies are expected to provide critical insights into the epigenetic fate of mobilized sequences as well as mechanisms of L1 regulation.

## Methods

### Mouse strains, insertion mapping, and bisulfite sequencing

Transgenic L1, *SBGFP*, and H1t-*SB100X* mouse strains are described in Supplemental Methods. Protocols for germ cell isolation, mouse genotyping, insertion mapping, and bisulfite sequencing analysis are detailed in Supplemental Methods. All primers are listed in Supplemental Table 1.

### CGI definition in masked and unmasked genomes

CGIs were predicted in the repeat-masked human (hg19/GRCh37) and mouse (mm9/NCBI37 and mm10/GRCm38) genomes using a local copy of newcpgseek from EMBOSS (Rice et al. 2000) at the default settings. Classically, CGIs are defined as regions of DNA that are >200 bp in length, >50% in GC content, and above 0.6 in the ratio of observed to expected CpGs (O/E ratio) (Gardiner-Garden and Frommer 1987). However, the biological significance of these parameters is still unclear (Illingworth and Bird 2009). The newcpgseek algorithm is agnostic of island length. Accordingly, we found that CGIs defined by newcpgseek encompassed islands of all lengths (Supplemental Fig. 5C). The vast majority of these CGIs had fulfilled the other two Gardiner-Garden and Frommer (1987) criteria (i.e., >50% in GC content and above 0.6 in O/E ratio) (Supplemental Fig. 5D). The UCSC Genome Browser uses the same algorithm to predict CGIs in the reference human and mouse genomes but it additionally filters the initial CGI set against all three Gardiner-Garden and Frommer (1987) criteria (Fujita et al. 2011). For the majority of our analyses, CGIs of ≥200 bp were used. To determine the contribution of retrotransposons to the CpG island landscape, CGIs were also predicted from the unmasked human genome (hg19/GRCh37) and islands that are ≥200 bp were selected for analysis. To define “repeat” versus “unique” islands, we compared the start and end coordinates between the islands predicted from the masked and unmasked genomes. If the start and end coordinates were identical between both genomes, the island was classified as “unique.” If the start and end coordinates were different in the unmasked (due to the presence of a repeat) or were only found in the unmasked genome, the island was categorized as “repeat.” To classify repeat CGIs, the start and end coordinates of CGIs and RepeatMasker annotated repeats (downloaded from the UCSC Genome Browser) were compared. A CGI is counted into one of the four types, depending on where the repeats landed within the CGI.

### Methylomes, methylation mapping, and slope calculation

Methylomes generated through unbiased whole-genome bisulfite sequencing (WGBS) approaches were utilized (Supplemental Table 2). Percentage methylation was calculated as a ratio of observed C/(observed C + observed T) × 100. Coverage was calculated for each CpG as well as for individual CGIs. On average, all CGIs had 5× coverage in the analyzed methylomes. Procedures for mapping methylation to CGIs and surrounding CpG sites, for calculating shore slopes, and for mapping differentially methylated regions are detailed in Supplemental Methods.

## Data access

Bisulfite sequencing data generated in this study have been submitted to the NCBI Trace Archive (http://www.ncbi.nlm.nih.gov/Traces/trace.cgi) under trace IDs (TI) 2342803997–2342805635. Custom Perl scripts used in this study are available as Supplemental Scripts.

## Supplementary Material

Supplemental Material
