# Supplementary material for: Retrotransposition creates sloping shores: a graded influence of hypomethylated CpG islands on flanking CpG sites
Source: Genome Res. 2015 Aug;25(8):1135–46. doi: 10.1101/gr.185132.114 (PMC4509998; doi:10.1101/gr.185132.114)
Supplement: Supplemental Material [file supp_25_8_1135__index.html]

Retrotransposition creates sloping shores: a graded influence of hypomethylated CpG islands on flanking CpG sites — Retrotransposition creates sloping shores: a graded influence of hypomethylated CpG islands on flanking CpG sites — Supplemental Material 

# Retrotransposition creates sloping shores: a graded influence of hypomethylated CpG islands on flanking CpG sites

## Supplemental Material

**Files in this Data Supplement:**

- Supplemental Figures.pdf
- Supplemental Methods.pdf
- Supplemental Tables.pdf
- Supplemental scripts.zip
