# Supplementary material for: Retrotransposition creates sloping shores: a graded influence of hypomethylated CpG islands on flanking CpG sites
Source: Genome Res. 2015 Aug;25(8):1135–46. doi: 10.1101/gr.185132.114 (PMC4509998; doi:10.1101/gr.185132.114)
Supplement: Supplemental Material [file supp_gr.185132.114_Supplemental_Figures.pdf]

Supplemental Figures

Supplemental Figure 1. DNA methylation profile of the *GFP* marker sequence in G0 animals. (**A**) The experimental workflow. A donor-containing animal is bred to a wild-type animal. The progeny is genotyped with a primer pair flanking the intron, shown in Figure 1A. Three genotypes are expected. The animal only carrying an insertion is designated as G0. It is further bred to a wild-type animal. Tissues from G0 and subsequent generations (G1, G2, etc) are collected and analyzed. (**B**) The complete nucleotide sequence for hopB1498. CpG dinucleotides are highlighted in uppercase letters. The region analyzed by bisulfite PCR is delimited by the two priming sites (underlined). (**C)** hopB1498. (**D**) hopB1712. (**E**) hop1718. (**F**) hopB1919. (**C-F**) Methylation dot plots for *GFP* in the heart, liver and testis of each G0 animal. The same data are summarized in a tabular form in Figure 1B. Each circle represents one CpG site along the *GFP* amplicon. Filled circles are methylated CpGs, open circles are unmethylated CpGs, and grey circles are uninformative sites due to sequencing error. Each row corresponds to the sequencing read from one cloned bisulfite PCR amplicon. HopB1712 and hopB1718 are identical in sequence and location but were carried in female and male siblings, respectively. No testis data are shown for hopB1712 and hopB1919 as both G0 animals are female.

Supplemental Figure 2. Dynamics of *GFP* methylation during male germ cell development. (**A**) Methylation dot plots of hopB1498 *GFP* at different postnatal time points. We observed that in the adult testis most bisulfite reads were devoid of methylation across the entire length and only a few reads were hypermethylated (Supplemental Fig.1E). Because the testis is composed of both somatic and germ cells, we reasoned that the unmethylated reads might have originated from testicular somatic cells. To test this hypothesis, we compared *GFP* methylation in hopB1498 G2 animals at three developmental time points: postnatal day 6 (P6), P20, and adult, when the proportion of germ cells increases to 1/6, 1/3, and >9/10 of the whole testis, respectively (Bellve et al. 1977). Indeed, the number of unmethylated bisulfite reads was largely consistent with the expected proportion of germ cells in the testis at each developmental stage: overall, about 81%, 34%, and 9% of the CpGs were methylated, respectively. (**B**) *GFP* methylation in heart, liver and testis across developmental time points for hopB1498. Unlike the testis, DNA methylation in the heart and liver remained high in all three time points. The same phenomenon was observed in G2 animals of hopB1712 and hopB1718 (see panels C,D). Testis data are reproduced from methylation dot plots in panel A. Each individual shape represents one biological replicate (i.e., a different animal). For each tissue type, the line represents the average among biological replicates. **(C)** *GFP* methylation through development for hopB1712. **(D)** *GFP* methylation through development for hopB1718. (**E**) The workflow for enrichment of testicular germ cells. To further demonstrate DNA methylation dynamics in the male germline, somatic and germ cells were also isolated from embryonic testes by fluorescence activated cell sorting (FACS) using the Pou5f1-*EGFP* transgene as a germ cell marker (Szabo et al. 2002). In a representative FACS plot, two cell populations are collected. Cells from Gate 2 are *GFP*-positive germ cells while cells from Gate 3 represent *GFP*-negative testicular somatic cells. (**F**) Bisulfite PCR primers are designed to specifically to amplify the retrotransposed *GFP* sequence. We used a forward primer (WA083) that anneals to the upstream of the splicing junction and a reverse primer (WA197) that is specific to the RSV promoter. The expected amplicon is 595 bp. The specificity of bisulfite PCR is tested with a panel of samples. The animal IDs are labeled on top of the gel. NTC, no template control. The molecule weight marker is the 1 Kb Plus DNA Ladder (Invitrogen). Three possible *GFP* alleles are present in these samples: (1) the intron-containing *GFP* allele from the donor L1 transgene, (2) the intronless *GFP* allele from L1 insertion(s), and (3) the Pou5f1-*EGFP* transgene. The genotype of each sample is indicated at the bottom of the gel (+/-, presence or absence of each allele). Sample B1395 was positive for both donor and insertion(s) in genotyping PCR, but only the insertion was amplified in this bisulfite PCR reaction. The expected intron-containing band would be 1495 bp. Sample O001 only contained the Pou5f1-*EGFP* allele and no amplification was detected as expected (no priming site for WA197). The intronless band was amplified from other samples carrying either a single germline insertion or both a germline insertion and the Pou5f1-*EGFP* allele. (**G**) Methylation dot plots of hopB1498 *GFP* in E14.5 embryos. (**H**) Methylation dot plots of hopB1498 *GFP* in E18.5 embryos. Four cell types (germ cell fraction, testicular somatic cell fraction, heart, and liver) are represented. Overall methylation (%) is listed on top of the each dot plot. HopB1498 was devoid of methylation in both E14.5 and E18.5 germ cell fractions, whereas high levels of methylation were observed in corresponding somatic cell fractions. **(I)** Methylation dot plots of hopB1718 *GFP* in E18.5 embryos. Testicular somatic and germ cell fractions are represented. Germ cell specific hypomethylation was also detected for hopB1718 in the E18.5 testis.

Supplemental Figure 3: Integrating the GPF marker by DNA transposon reproduces somatic-high, germ-cell-low methylation pattern. **(A**) Schematic of the *SBGFP* transgene. In the donor mouse, the transgene is present in a ~40-copy tandem array. The structure mirrors the *ORFeus* insertion, containing an intronless *GFP*

reporter cassette and a small portion of the 3' end of ORF2 (blue box in diagram). Inverted terminal repeats (ITR) from the SB system (Mates et al., 2009) flank each end. The black bar below *GFP* indicates the region amplified for methylation analysis (identical to that in Figure 1A). **(B)** The workflow. The high-copy *SBGFP* donor is first crossed to H1t-*SB100X*. Doubly transgenic male progeny is then crossed to a WT female to produce animals carrying a single-copy *SBGFP* insertion (designated as G0). Lineages with single-copy *SBGFP* insertions were mapped, segregated from the H1t-*SB100X* driver, if necessary, and bred for further analysis. **(C)** Methylation dot plots for adult liver and testis from two G0 founder animals. JR32 and JR33 each carry a single *SBGFP* insertion at different genomic locations. Percent methylation is stated on the right of each dot plot; each circle represents one methylated (filled) or unmethylated (open) CpG. Each row represents one sequenced bisulfite PCR amplicon. **(D,E)** Methylation in the liver and testis across generations for two *SBGFP* insertions: jump32 and jump33 are derived from G0 animals JR32 and JR33, respectively. Each data point represents a different biological replicate (animal). Line represents the average methylation among replicates. Data for G0 are reproduced from panel C.

Supplemental Figure 4: DNA methylation of CpG sites flanking the *GFP* insertion. (A) Schematic of CpGs at the 5' and 3' flank of the hopB1498 insertion. Scale represents distance of the CpG from the end of the *GFP* CGI. Points in black represent those investigated by this study; points in gray represent CpGs that were too distant to be successfully amplified due to the fragmented nature of bisulfite treated DNA. The insertion itself is colored in blue. The coordinates represent distances from the *GFP* CpG island contained within the insertion. The coordinate of the 5' or 3' boundary of the insertion (i.e., the distance from the *GFP* CGI) is marked in red. Note the length of poly(A) tract is not included when the distance is calculated. If the poly(A) is included the distances would increase by 17-30 bp. **(B,C)** Methylation dot plots for empty and filled alleles in three hopB1498 G1 animals. Filled circles represent methylated CpGs; open circles represent unmethylated CpGs. The liver (B) and testis (C) data are used to create graphs in Figure 2B and Figure 2C, respectively. Animal IDs are shown to the left. Location of the insertion is shown with a triangle. An open triangle marks the insertion site in the empty allele; a filled triangle marks that in the filled allele. **(D)** Schematic of CpGs in the 3' flank of hopB1718 insertion. **(E)** Schematic of CpGs in the 3' flank of hopB1919 insertion.

Supplemental Figure 5: Computational analysis of sloping CGI shores in human methylomes. (A,B) Analysis of low (A) and high (B) sperm CGIs using a smaller interval size of 100 bp (Interval size of 250 bp is used in the main text). Raw data used are identical to those in Figure 3C. Intervals are marked on the x-axis with the starting and ending position of the bin. **(C)** Distribution of CGI lengths. CGIs are defined by the newcpgseek algorithm. Percentage reflects proportion in the total number of CGIs. **(D)** Regardless of length, the majority of CGIs identified by newcpgseek fulfills two other CGI criteria proposed by Gardiner-Garden & Frommer. CGIs are first separated into three length groups. Note Gardiner-Garden & Frommer define CGIs as clusters of CpGs that are >200 bp in length, >50% in GC content, and >0.6 in O/E ratio. CGIs within each length group are then separated into two groups according to either the GC content cutoff or the O/E ratio cutoff. **(E)** Analysis of the effect of CGI length variation on the slope of single, low CGIs in sperm. Each line represents one CGI size range (see the associated legend to the right). Error bars represent standard errors of the mean. **(F)** Equivalency of global and local averaging approaches for visualizing sloping shores. Low CGIs in sperm methylome are used as an example. Local averaging data are reproduced from Figure 3C. Error bars represent standard errors of the mean. **(G)** Equivalency of global and local averaging approaches for slope calculation. Low CGIs in colon and sperm methylomes are used as examples. Two approaches yield nearly identical slopes. Error bars represent standard deviations (SDs), which can be calculated by using the local approach. Here we chose to display SDs so that data variation can be visually appreciated. The corresponding standard errors (SEs) are very small (0.092-0.125 for colon, and 0.083-0.139 for sperm) as the number of CGIs is vast in our data sets (10,259 for colon, and 13499 for sperm).

Supplemental Figure 6: Sloping shores in mouse methylomes. (A) Percent of total CGIs that fall into each classification for mouse liver and sperm methylomes. Total CGIs are defined from the repeatmasked mouse genome. The classification is identical to those in Figure 3A. **(B,C)** Sloping shores for low (B) and high (C) CGIs in mouse liver (Hon et al., 2013) and sperm from two different mouse strains: C57BL/6N (Kobayashi et al., 2012) and DBA/2J (Wang et al., 2014). The slope of shore is given in table to the right. Each point represents the bin average for that interval, as in Figure 3C. Tissues are color-coded in panels B and C but legends are drawn only in panel C.

Supplemental Figure 7: CGI shores in human tissues and cell lines. (A,B) Slope of shores for high and low CGIs for analyzed human tissues (A) and cell lines (B). The table of low CGIs for human tissues is reproduced

from Figure 3F. **(C,D)** Comparison of high CGIs (C) and low CGIs (D) between colon and colon tumor from the same individual. **(E,F)** Differentially methylated regions between human tissues (E) and cell lines (F). Briefly, DMRs are mined by comparing shores between low CGIs present in the two tissues and taking the cumulative difference between CpGs. Pairwise comparisons are indicated in the color-coded legend. A positive value indicates that the first tissue is more methylated while a negative value indicates the second tissue is more methylated. The x-axis designates ranges of percent cumulative difference. **(G)** Example of tDMR between liver and hippocampus. Percent cumulative difference is listed above plot. Each dot represents the methylation level of one CpG site. Plot does not include CpGs within the CGI itself. **(H)** Example of the lack of tDMR between liver and colon. The genomic location is identical to that panel G. **(I)** cDMR between colon and matched tumor. Location is different from panels G and H.

Supplemental Figure 8: Clustered CGIs in ORFeus transgene. (A) Schematic of ORF1 and ORF2 segments contained in the hopB1919 insertion. Amplicons for bisulfite PCR are designated by the black bars below. **(B)** Location of CGIs in the ORF1/ORF2 region as defined by newcpgseek. The CGIs with length >200 bp are numbered 1 to 5. **(C)** Average Methylation of selected regions in the heart of three G1 animals carrying the hop1919 insertion. CpGs belonging to defined CGIs are colored in gray. Error bars designate standard errors of the mean. **(D)** Methylation in the liver. **(E)** Methylation in the testis. Note more regions were analyzed in the testis to reveal the transition in methylation levels.

Supplemental Figure 9: **Crosstalks between neighboring CGIs in human sperm methylome. (A-G)** Pairs of CGIs at different distances in the human sperm methylome. Plot represents global average over 100 bp intervals, as in Figure 4. The x-axis designates distance from first CGI (on the left). Error bars represent standard errors of the mean. All graphs are drawn to the same scale. (A) 5000-6000 bp apart, reproduced from Figure 4C. (B) 4000-5000 bp apart. (C) 3000-4000 bp apart. (D) 2000-3000 bp apart, reproduced from Figure 4D. (E) 1500-2000 bp apart. (F) 1000-1500 bp apart. (G) 500-1000 bp apart, reproduced from Figure 4E. The slope of a hypomethylated CGI is significantly affected by another CGI if they are located within ~3000 bp from each other.

Supplemental Figure 10: Crosstalks between neighboring CGIs in human liver methylome. (A) Pairs of CGIs between 5000-6000 bp apart in human liver. Plot represents global average over 100 bp intervals as in Figure 4. Error bars represent standard errors of the mean. The x-axis represents distance from first CGI on the left and is drawn to scale among panels A-C. Slope of the shore at defined positions is represented on the table below the corresponding plot. **(B)** Pairs of CGIs between 2000-3000 bp apart with corresponding slopes. **(C)** Pairs of CGIs between 500-1000 bp apart with corresponding slopes.

Supplemental Figure 11: Dynamics of CGI methylation through reprogramming. (A) Average methylation of low and high CGIs through embryonic development. Low and high assignment was made based on the methylation status of each CGI in E7.5 embryo. Error bars represent standard errors of the mean. **(B)** Average methylation of low and high CGIs through male germ cell development. Low and high assignment was made based on the methylation status of each CGI in sperm. Error bars represent standard errors of the mean.

Supplemental Figure 12: Repeat and unique CGIs show similar sloping shores. Average methylation of CpGs in 250 bp bins within 5 kb of a CGI in colon and sperm. Each point represents a bin average. Error bars represent standard errors of the mean. Intervals are marked by the starting coordinate, which indicates the distance of interval from the CGI; positive numbers are downstream, negative numbers are upstream of the CGI. (A) Low CGIs in colon. (B) High CGIs in colon. (C) Low CGIs in sperm. (D) High CGIs in sperm.

Supplemental Figure 13: Lack of methylation in somatically acquired insertions. (A) Methylation dot plots of methylation in the *GFP* marker for somatic insertions in the heart and liver from adult donor-containing animals. **(B)** Methylation dot plots in the *GFP* marker for somatic insertions in the heart and liver from donor-containing E14.5 embryos. On average, somatic retrotransposition frequency is ~0.4 per cell. The insertions were analyzed as a pool. Each row may represent a unique insertion or a different amplicon from the same insertion. Overall methylation is listed on the top of each plot.

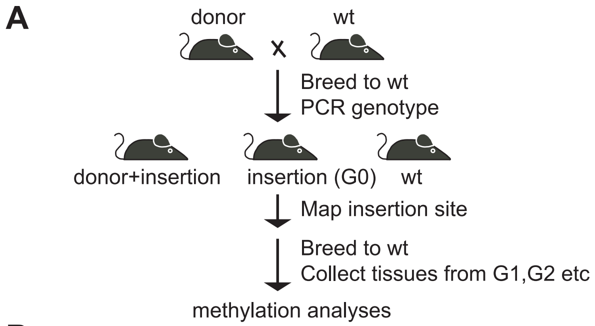

B

aagaaagtgttgtgCGagttcatgaagttcctggccaagtggatggacctggagagcatcatcctgagCGaggtgacccagagccagCGcaacagccaca
acatgtacagcctgatcagCGgctactagcctaaaacCGgtgatacccaCGatataagatacaattgcctaaacacatgaaactcaagaaaaatgaagac
tgaagtgtggacactatgcccctccttagaagtgggaacaaaacagccatagaaagagttacagaaacaaagtttggagctgagatgaaaggagagacca
tgtagaaactgccatatccagggttccaccccataatcagcatccaagctttgacaccattgcatatactaggaagattttatCGaaaggacccagatgt
agctgtctcttgtgagactatgcCGggcctagcaacacagaagtggatgctcacagtcagctatggatggatcacagggctttcaatggaggagctaga
gaaagtacccaaggagctaaagggatctgcaactctataggtggaacaacattatgagttatcagcctgctttttgtacaaacttatcccCGtCGaCGg
atCGatcCGaacaaaCGacccaacacccCGtgCGttttattctgtctttttattgcCGatcccctcagaagaactCGtcaagaaggCGatagaaggCGatg
CGctgCGaatCGggagCGgCGatacCGtaaagcaCGaggaagCGgtcagcccattCGcCGccaagctcttcagcaatatcaCGggtagccaaCGctatgt
cctgatagCGgtCGgcCGctttacttgtacagctCGtccatgcCGtgagtgatcccGgCGgCGgtcaCGaactccagcaggaccatgtgatCGCGcttct
CGttggggtctttgctcagggCGgactgggtgctcaggtagtggttgtCGggcagcagcaCGgggcCGtCGcCGatggggtgttctgctggtagtggtc
ggccagctgcaCGctgcCGtcctCGatgttgtggCGgatcttgaagttggccttgatgcCGttcttctgcttgtCGgCGgtgatatagaCGttgtggctg
ttgtagttgtactccagcttgtgccccaggatgttgcCGtcctccttgaagtCGatgcccttcagctCGatgCGgttcaccagggtgtCGccctCGaact
tcacctCGgCGCGggtcttgtagttgcCGtCGtccttgaagctgatggtgCGctcctggaCGtagccttCGggcatggCGgacttgaagaagtCGtggCG
cttcatgtggtCGgggtagCGgctgaagcactgcaCGcCGtagctgaaggtggtcaCGagggtgggccagggcaCGgcagcttgcCGgtggtgcagatg
aacttcagggtcagcttgcCGtaggtggcatCGccctCGccctCGcCGgacaCGctgaacttgtggcCGtttaCGtCGcCGtccagctCGaccaggatgg
gcaccacccCGgtgaacagctcctCGcccttgctcaccatggtggctgctagcaatgtggtgaatggtcaaatggCGtttattgtatCGagctaggcact
taaatacaatatctctgcaatgCGgaattcagtggttCGtccaatccatgtcagacccCGtctgttgccttcctaataaggcaCGatCGtaccaccttact
tccaccaatCGgcatgcaCGgtgctttttctctccttgtaaggcatgttgctaactcatCGttaccatgttgcaagactacaagagtattgcataagact
acatttccccctccctatgcaaaagCGaaactactatatcctgaggggactcctaacCGCGtacaacCGaagcccCGctggggaccactttgtacaagaa
agctgggtcctaggtCGgatccttttccctctgccaaaaattatggggacatcatgaagccccttgagcatctgacttctggctaataaaggaaatttat
tttcattgcaaaaaaaaaaaaaaaaaaaaaaaaaaaaaaaaaaaaaaaaaaaaaaaaaaaaaaaaaaaaaaaaaaaaaaaaaaaaaaaaaaaaaa
aaaaaaaaaaaaaaagaaagtgttgtg

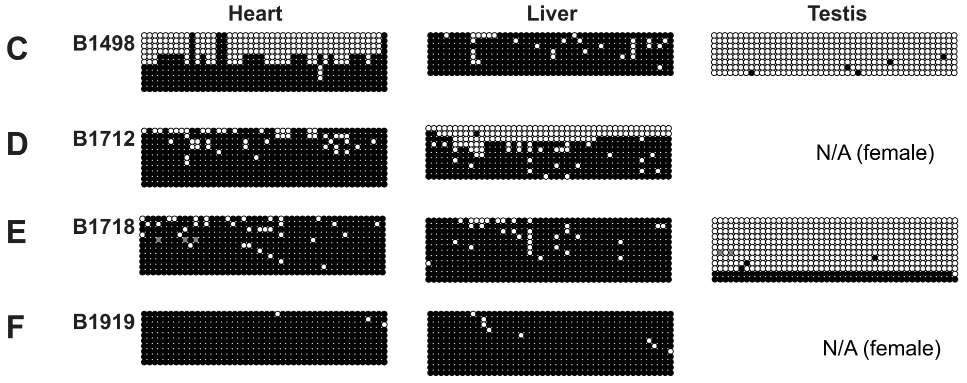

Supplemental Figure S1

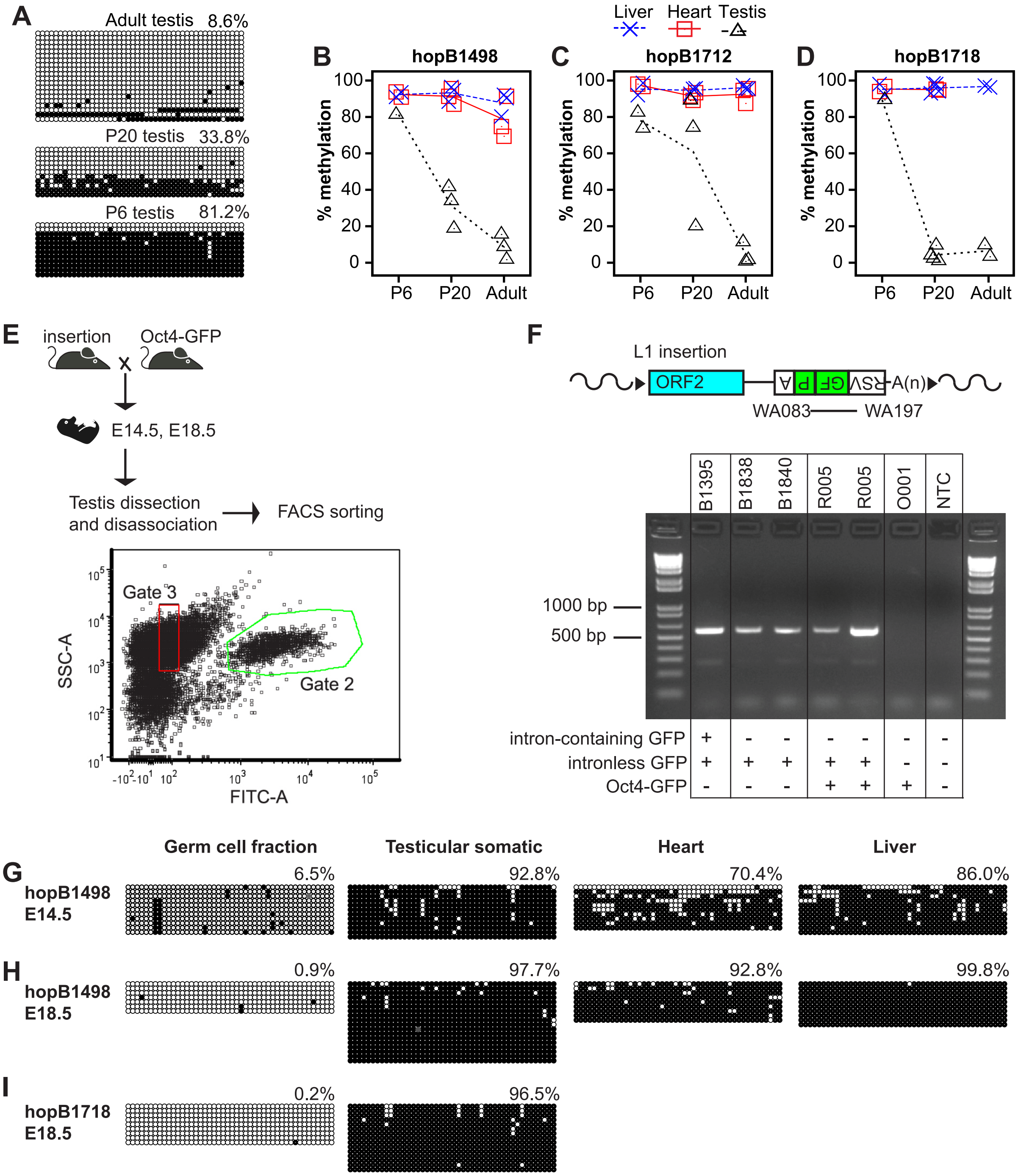

Supplemental Figure S2

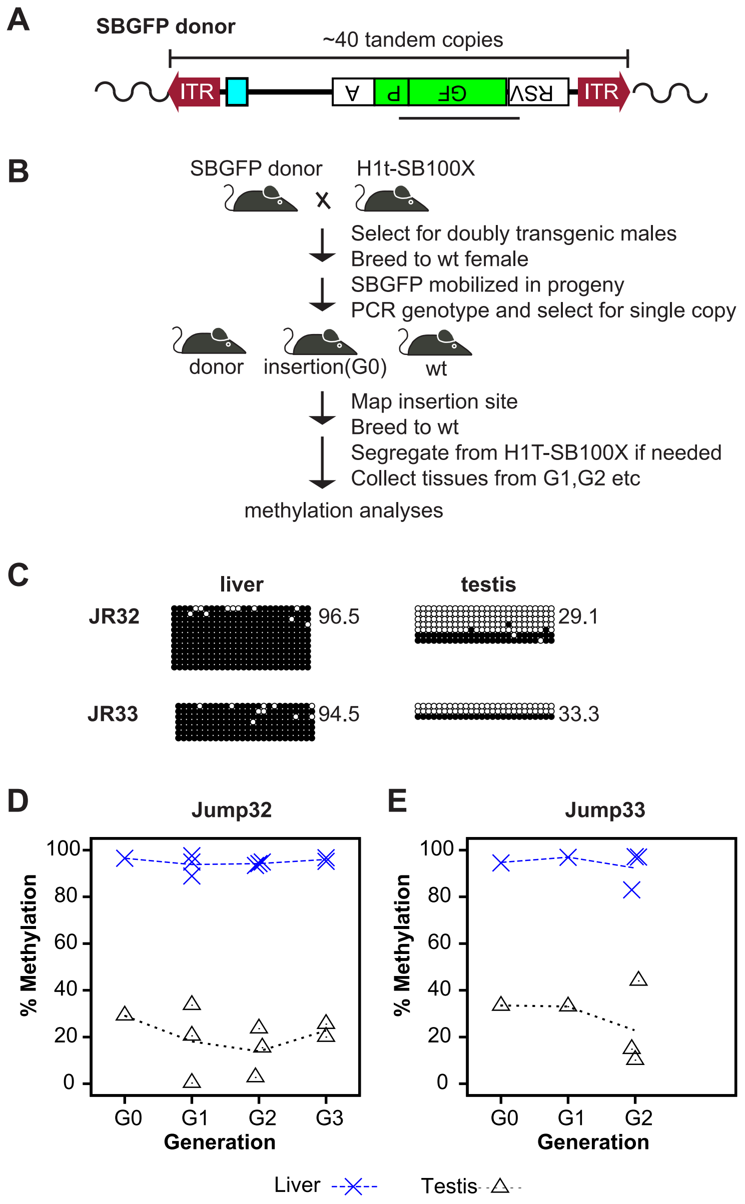

Supplemental Figure S3

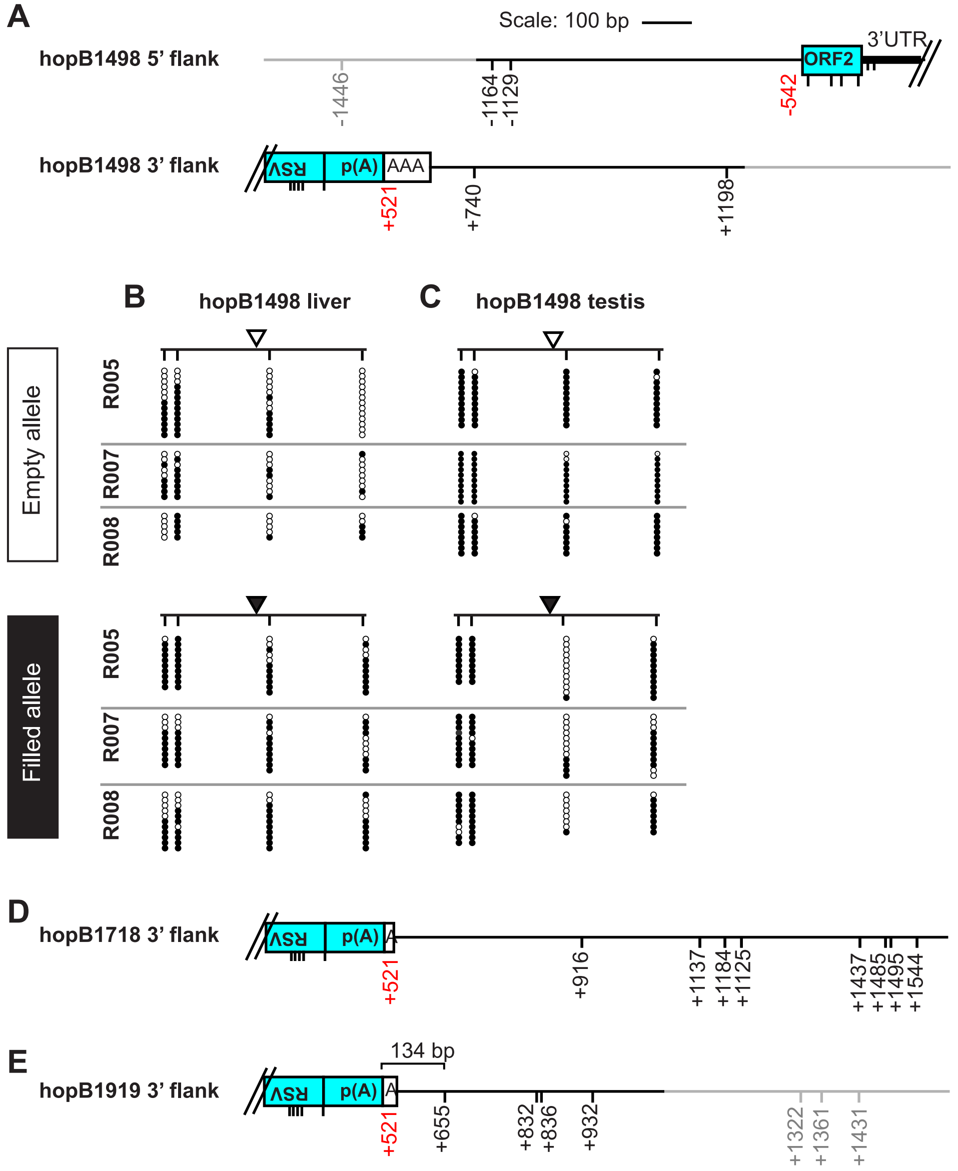

Supplemental Figure S4

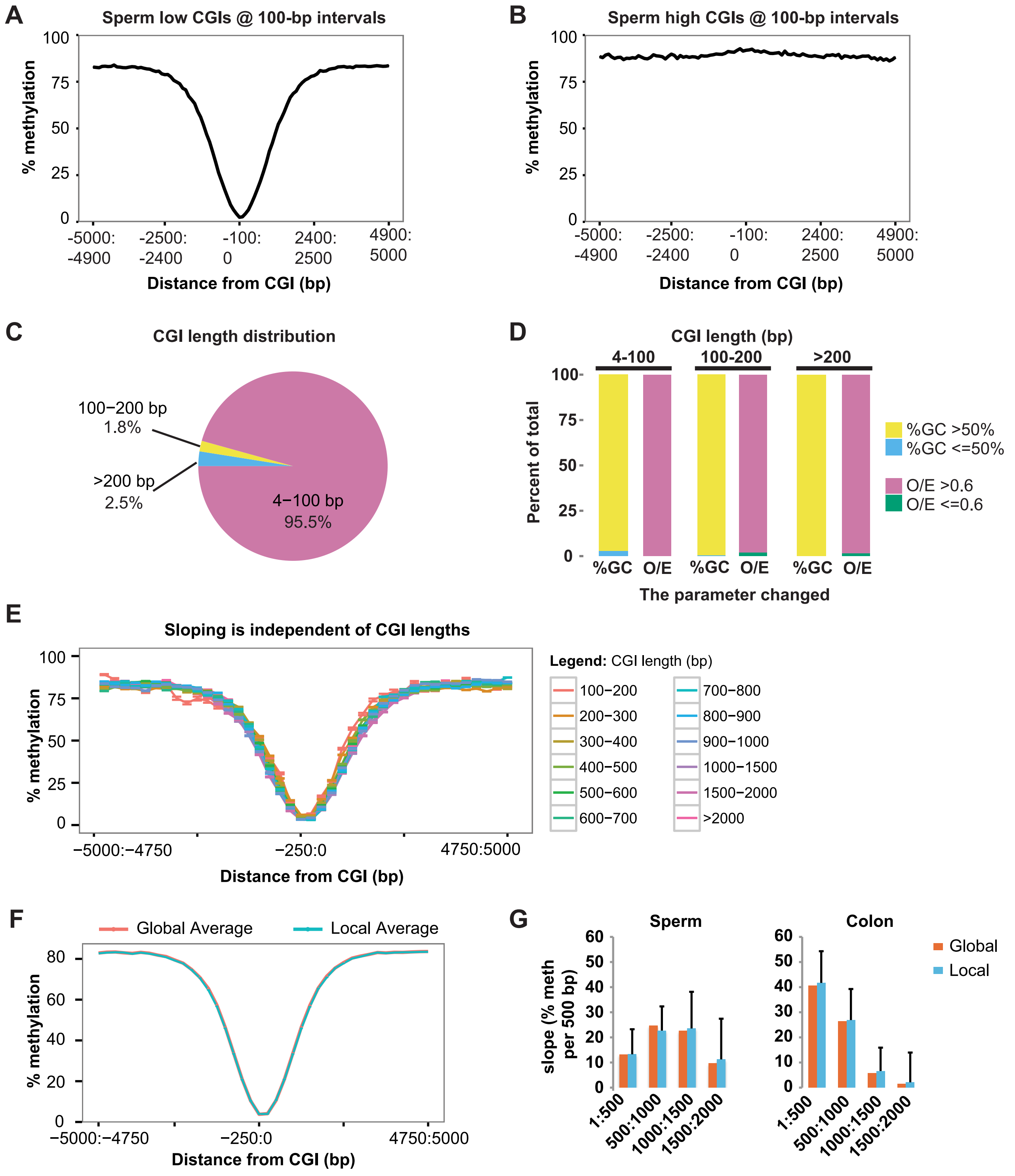

Supplemental Figure S5

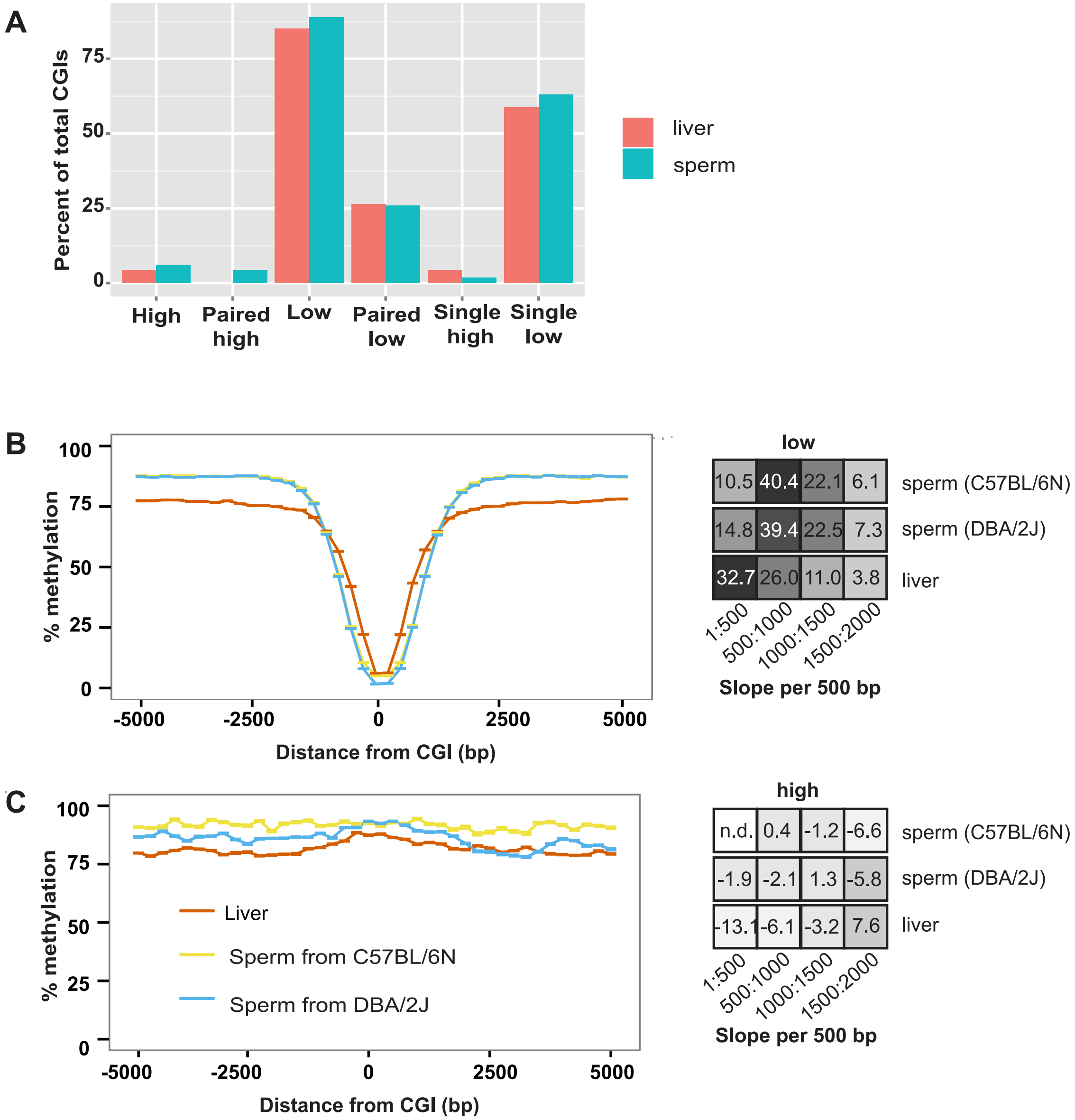

Supplemental Figure S6

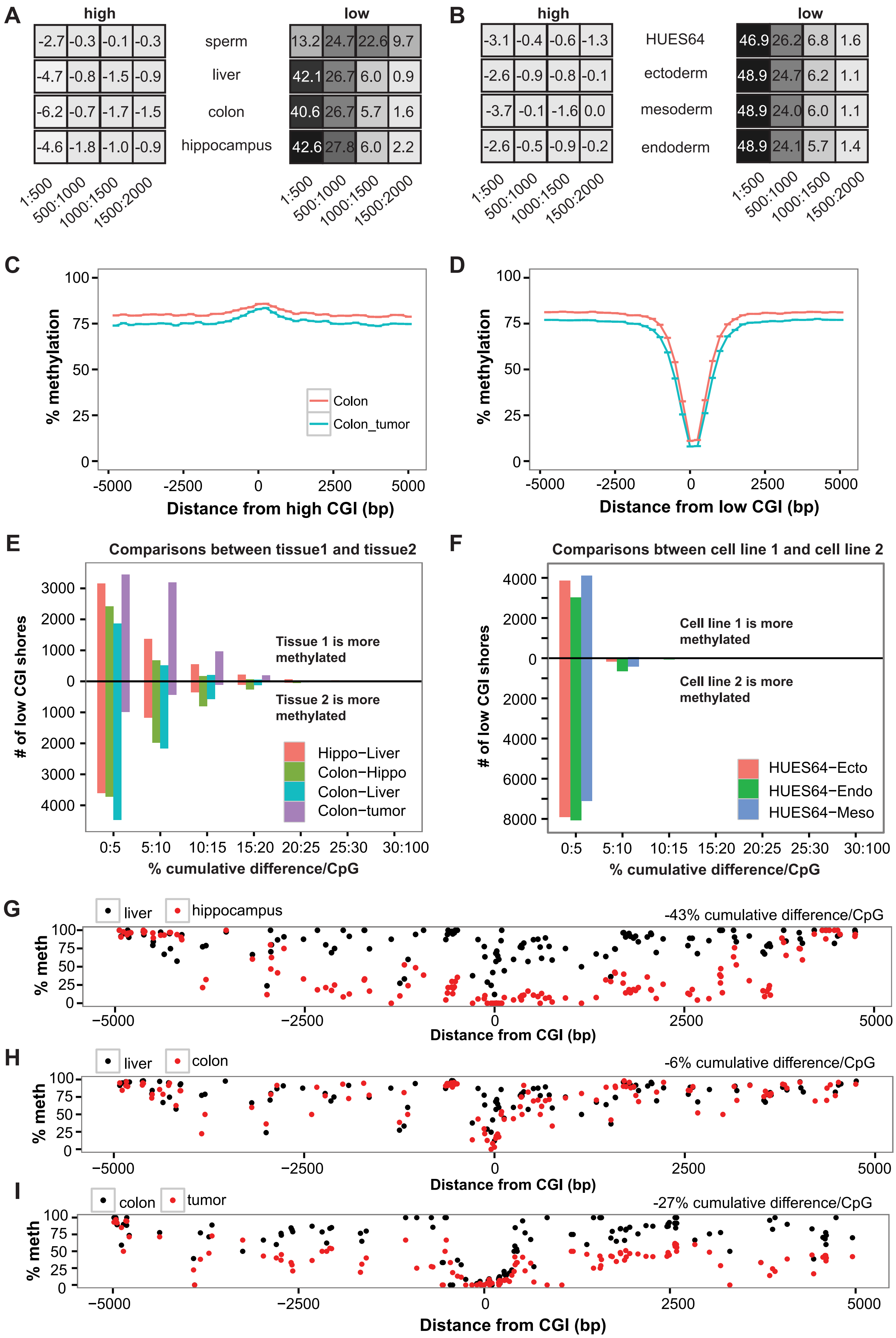

Supplemental Figure S7

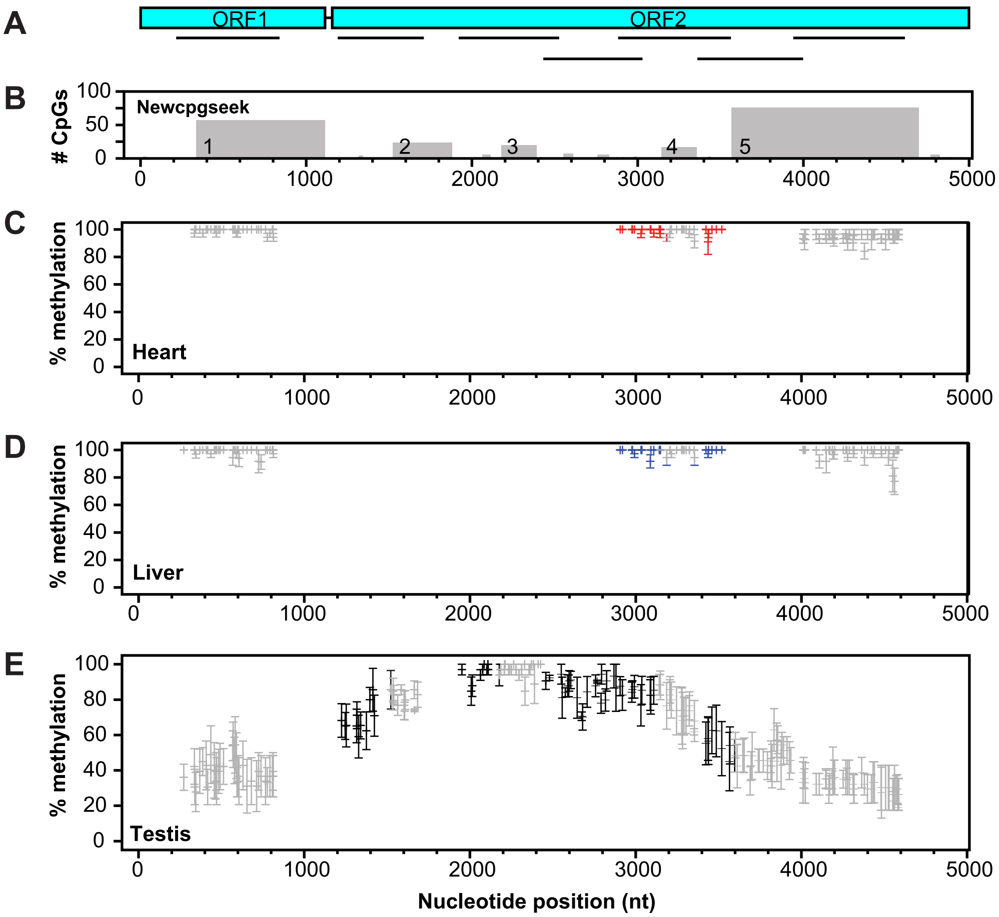

Supplemental Figure S8

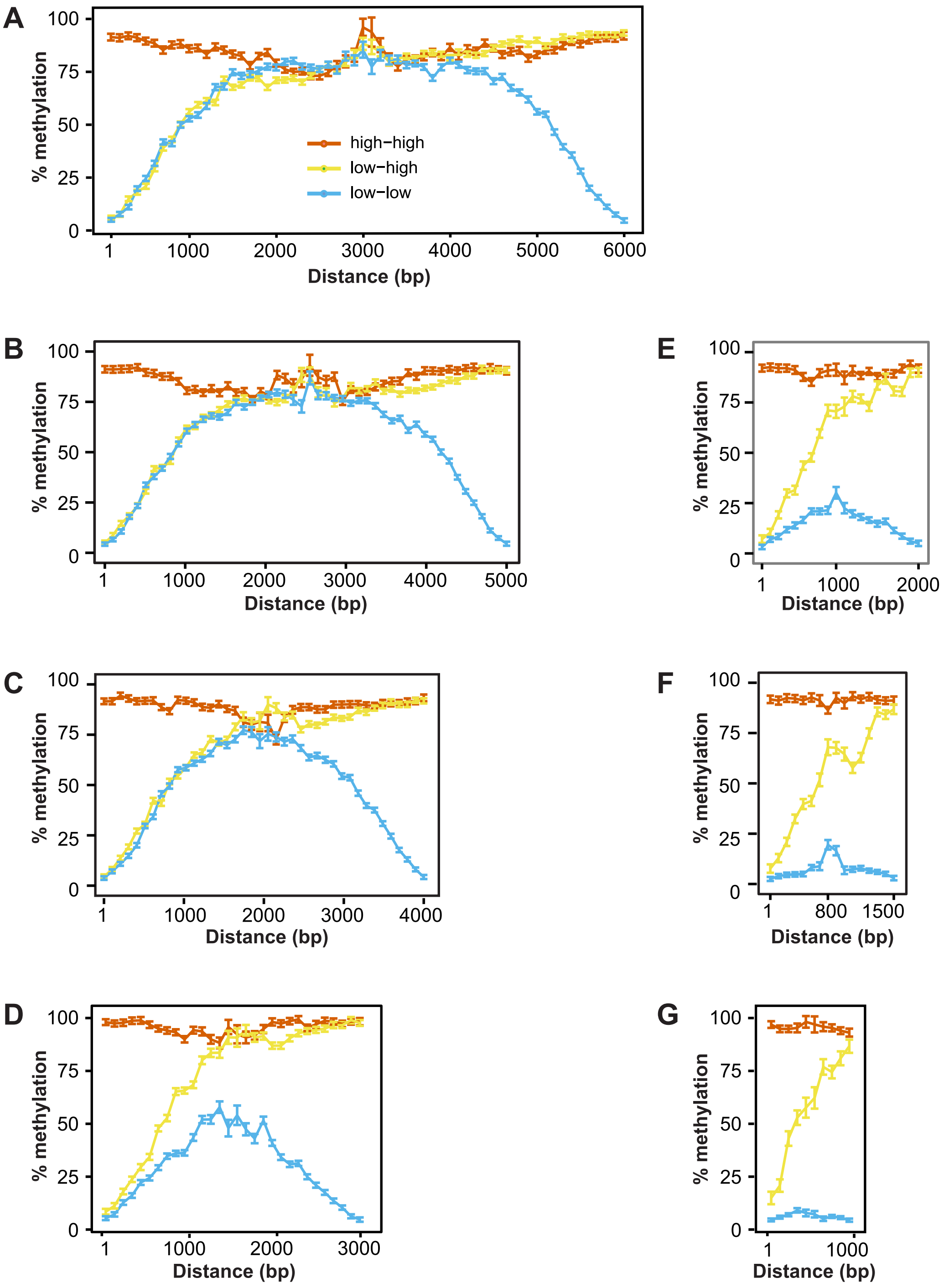

Supplemental Figure S9

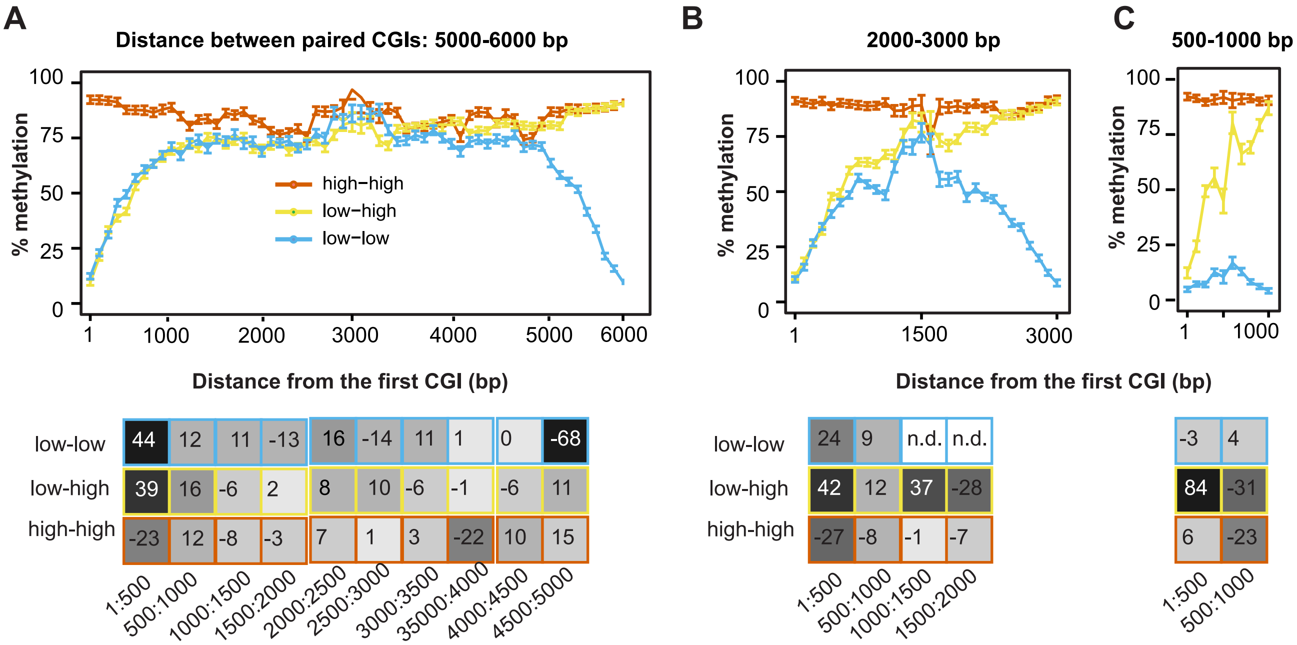

Supplemental Figure S10

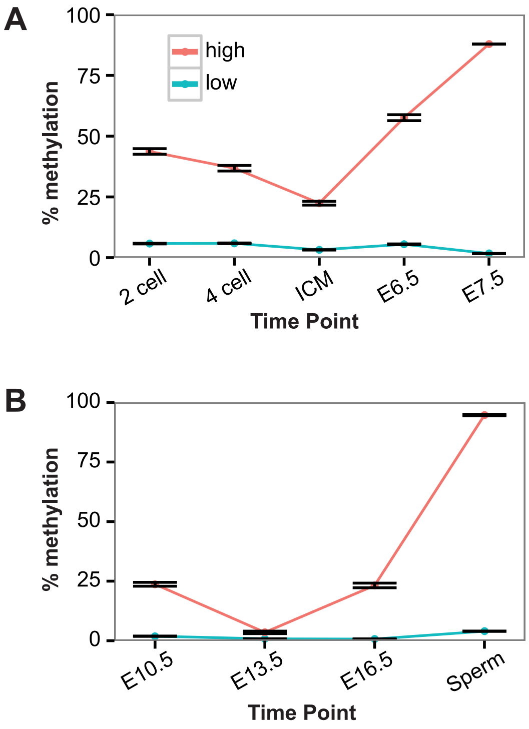

Supplemental Figure S11

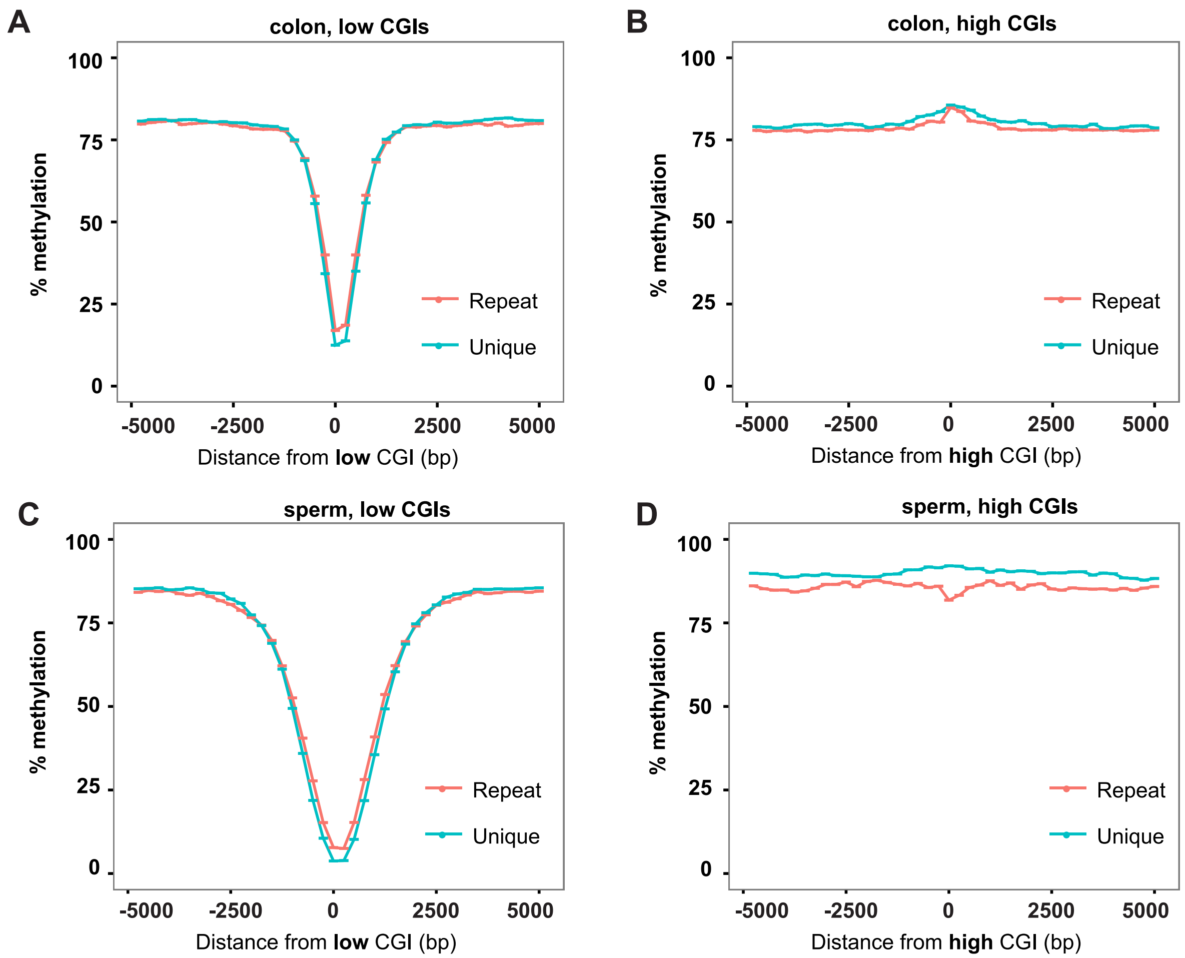

Supplemental Figure S12

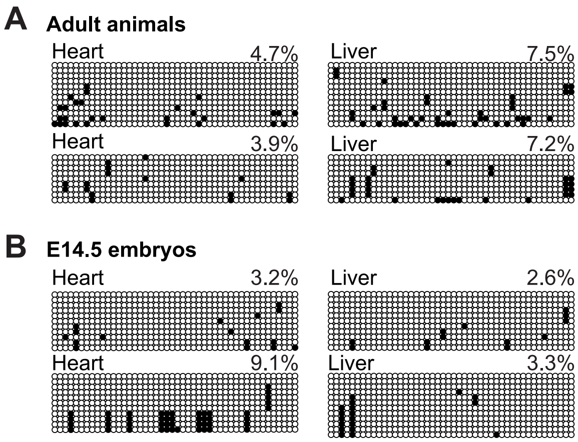

Supplemental Figure S13
