# Supplementary material for: Retrotransposition creates sloping shores: a graded influence of hypomethylated CpG islands on flanking CpG sites
Source: Genome Res. 2015 Aug;25(8):1135–46. doi: 10.1101/gr.185132.114 (PMC4509998; doi:10.1101/gr.185132.114)
Supplement: Supplemental Material [file supp_gr.185132.114_Supplemental_Methods.pdf]

Supplemental Methods

Mouse lines and tissues
This study was performed in strict accordance with the standards in the Guide for the Care and Use of Laboratory Animals of the National Institutes of Health and was approved by the Institutional Animal Care and Use Committee. Transgenic L1 mice (An et al. 2006) were housed in a specific-pathogen-free vivarium and maintained in the C57BL/6J (B6) background unless indicated otherwise. Transgenic *SBGFP* mice were produced by pronuclear microinjection of C3HxB6F2 embyos at the Gene Targeting and Transgenic Resource Facility, Roswell Park Cancer Institute. A male *SBGFP* founder mouse (ID 8351) was identified and backcrossed to wild-type B6. The progeny were crossed to H1t-*SB100X* transgenic mice (Supplemental Fig.3B). H1t-*SB100X* mice were produced in the C56BL/6N background at Max Delbrück Center for Molecular Medicine and will be detailed elsewhere. Pou5f1-*EGFP* transgenic mice were acquired from the Jackson Laboratory (Stock number 004654) (Szabo et al. 2002). Genomic DNA was isolated from tail biopsies using the Gentra PureGene Tissue Kit (Qiagen). For methylation analysis, mouse tissues were dissected at specific embryonic or postnatal ages as indicated and stored in RNAlater (Sigma) until further processed. Adult tissues were collected from animals at 7–10 weeks of age.

Germ cell isolation
To obtain embryonic time points, timed pregnancies were achieved by separating the breeding pair when a vaginal plug was observed. Embryonic testes were dissected and placed in ice-cold 1x phosphate buffered saline (PBS). Control embryonic heart and liver tissues were collected in RNAlater. Testicular cells were disassociated using a modified germ cell disassociation protocol (Buehr and McLaren 1993). Briefly, testes were repeatedly pipetted in 250 µL of 0.25% trypsin with EDTA and incubated for 2–3 min at 37°C. Cells were further disassociated with one more round of pipetting followed by another 2–3 min incubation. Disassociation was confirmed under an inverted microscope. One hundred microliters of fetal bovine serum was added to inactivate the trypsin. Cells were then filtered through a 40 µm filter, spun down at 600 x g for 7 minutes, and resuspended in 500 µL of Dulbecco's PBS with serum (DPBS-S) and placed on ice. *GFP*-positive and negative fractions were collected with a FACS Vantage SE cell sorter (Beckman Dickson), spun down, resuspended in 10 µL of 1x PBS, and used for bisulfite sequencing.

Genotyping and insertion mapping
PCR genotyping were performed as previously described (An et al. 2006). The 3′ flanking sequence for each L1 insertion was mapped by either inverse PCR or splinkerette PCR as previously described (An et al. 2009; Grandi et al. 2013). The 5′ genomic junction was subsequently determined by inverse PCR with L1 primers that are complementary to the 5′ end of the L1 sequence. The insertion length was estimated for hopB1919 by genotyping with sets of PCR primers along the entire length of the donor transgene. All primers are listed in Supplemental Table 1.

Bisulfite sequencing analysis
For bisulfite sequencing, genomic DNA was isolated with either the Gentra PureGene Tissue Kit or the DNeasy Blood and Tissue Kit (Qiagen). Tissues were homogenized using a TissueLyser II (Qiagen). Bisulfite treatment was conducted with either the EpiTect Plus DNA Bisulfite Kit or EpiTect Plus LyseAll Bisulfite Kit (Qiagen). PCR amplification, gel extraction, TA cloning, and sequence analysis were performed as previously described (Rosser and An 2010). Briefly, targeted regions were amplified with either Ex-Taq or Epi-Taq (Takara) using bisulfite specific primers (Supplemental Table 1). Desired amplicons were gel extracted using the QIAquick Gel Extraction Kit (Qiagen) and clone into the TA vector (Stratagene). White bacterial colonies were picked for Sanger Sequencing. DNA sequences were aligned to the reference target using MacVector and DNA methylation status was analyzed using QUMA (Kumaki et al. 2008). Methylation plots were generated by QUMA and downloaded. Data were visualized with a command-driven interactive function-plotting program, gnuplot version 4.6 (http://gnuplot.info/).

Mapping methylation to CpG islands
Methylomes generated through unbiased whole genome bisulfite sequencing (WGBS) approaches were utilized (Supplemental Table 2 for accession numbers). Processed CpG methylation data were downloaded from NCBI GEO (Barrett et al. 2013). Using the newcpgseek CGI definitions, methylation levels of individual CpGs were mapped onto the genomic intervals corresponding to each island using a custom perl script (Supplemental scripts: "CGImapper.pl"). The average and standard deviation of methylation (n-1) were calculated for each island. A custom script was then used to segregate high vs. low CGIs (Supplemental scripts: "high_low_split.pl"), where high CGIs were defined as those with an average methylation of 80% or greater and low CGIs as those with an average methylation of 20% or lower. A custom script was used to categorize CGIs as single or paired, depending on the distance of their nearest CGI neighbor (Supplemental scripts: "Single_CGI_finder.pl" for singles and "CGI_pair_finder.pl" for paired). Those with neighbors within 10kb on either end of the interval were designated paired while those with neighbors further than 10kb from either end were designated single. To check for neighbors, a list of all CGIs >200 bp in length was used regardless of methylation status. For individual cell and tissue types, the high/low CGI calling was made in reference to each methylome. For tracing sloping shore dynamics through development, high/low CGI calling was based on the eventual methylome (E7.5 for embryonic and sperm for germ cell development) and those same sets of CGIs were traced through developmental time points. For the unmaked genome, single CGIs were predicted using the full complement of unmasked CGIs.

Mapping methylation to surrounding CpG sites
Surrounding CpG methylation for the shores of the targeted islands was calculated using a custom script (Supplemental scripts: "CGI_surrounding.pl" for single and "CGI_pair_surrounding.pl" for paired"). For single CGIs, each CGI shore was binned into either 100 bp or 250 bp intervals, and the average methylation for that interval was calculated (see below). Then, all of the intervals were aggregated to produce the overall interval averages (Supplemental scripts: "local_average.pl"). For the paired CGIs, a global approach was used, as many shores did not have many CpG data points (Supplemental scripts: "global_pairs.pl"). All plots were visualized using the ggplot2 package for R (Wickham 2009).

Calculating shore slopes with local or global averaging approaches
For local averages, each CpG island was treated uniquely and the average methylation of all CpGs surrounding that island within the 250 bp bin was calculated. These averages were then averaged to get the genome wide average. For the global average, all CpGs were treated as if they belonged to the same CpG island and were binned into 250 bp intervals according to their distance from the CGI. The average of each bin was then calculated. Both approaches give the same global picture, but the local averages represent a processing scheme that is more biologically relevant as each CGI is treated as its own entity. Local averaging was used for all single CGIs analyzed. Global averaging was used for paired CGI analysis and to determine shore slopes. The slope, or the rate of methylation change in sloping shores, was calculated for a span of 500 base pairs over four intervals: 1-500, 500-1000, 1000-1500 and 1500-2000. The process is graphically depicted in Fig.3E. The beginning and ending methylation levels for each 500 bp span were calculated from the first 10 bp of each span, including bp 1-10, 501-510, 1001-1010, 1501-1510 and 2001-2010. The difference between each interval was then calculated using a custom script (Supplemental scripts: "slope_calc.pl"). For single CGIs, upstream and downstream CpGs were combined. Nearly identical slopes were obtained when calculated by global v. local averaging approaches (Supplemental Figure 5G).

Mapping differentially methylated regions
To find DMRs, a common set of low CGIs were obtained for the two tissues to be analyzed. Then, the difference in methylation for each CpG in the shore was calculated using a custom Galaxy workflow (Goecks et al. 2010). The cumulative difference between all CpGs in the shore was calculated and normalized by dividing by the total number of CpGs in the shore using a custom script (Supplemental

scripts: “Shore_DMRs.pl” and “Shore_DMRs_sum.pl”). For example, if the cumulative difference for a shore of 150 CpGs was 500, the cumulative difference per CpG would be 500/150. Results were plotted using a local copy of R version 2.15.3 (R Core Team 2014).

References

An W, Davis ES, Thompson TL, O'Donnell KA, Lee CY, Boeke JD. 2009. Plug and play modular strategies for synthetic retrotransposons. *Methods* **49**(3): 227-235.

An W, Han JS, Wheelan SJ, Davis ES, Coombes CE, Ye P, Triplett C, Boeke JD. 2006. Active retrotransposition by a synthetic L1 element in mice. *Proceedings of the National Academy of Sciences of the United States of America* **103**(49): 18662-18667.

Barrett T, Wilhite SE, Ledoux P, Evangelista C, Kim IF, Tomashevsky M, Marshall KA, Phillippy KH, Sherman PM, Holko M et al. 2013. NCBI GEO: archive for functional genomics data sets--update. *Nucleic acids research* **41**(Database issue): D991-995.

Buehr M, McLaren A. 1993. Isolation and culture of primordial germ cells. *Methods Enzymol* **225**: 58-77.

Goecks J, Nekrutenko A, Taylor J, Galaxy T. 2010. Galaxy: a comprehensive approach for supporting accessible, reproducible, and transparent computational research in the life sciences. *Genome biology* **11**(8): R86.

Grandi FC, Rosser JM, An W. 2013. LINE-1 derived poly(A) microsatellites undergo rapid shortening and create somatic and germline mosaicism in mice. *Mol Biol Evol* **30**(3): 503-512.

Kumaki Y, Oda M, Okano M. 2008. QUMA: quantification tool for methylation analysis. *Nucleic acids research* **36**(Web Server issue): W170-175.

R Core Team. 2014. R: A Language and Environment for Statistical Computing. R Foundation for Statistical Computing, Vienna, Austria.

Rosser JM, An W. 2010. Repeat-induced gene silencing of L1 transgenes is correlated with differential promoter methylation. *Gene* **456**(1-2): 15-23.

Szabo PE, Hubner K, Scholer H, Mann JR. 2002. Allele-specific expression of imprinted genes in mouse migratory primordial germ cells. *Mech Dev* **115**(1-2): 157-160.

Wickham H. 2009. *ggplot2: Elegant Graphics for Data Analysis*. Springer-Verlag New York.
