# Supplementary material for: Retrotransposition creates sloping shores: a graded influence of hypomethylated CpG islands on flanking CpG sites
Source: Genome Res. 2015 Aug;25(8):1135–46. doi: 10.1101/gr.185132.114 (PMC4509998; doi:10.1101/gr.185132.114)
Supplement: Supplemental Material [file supp_gr.185132.114_Supplemental_Tables.pdf]

Supplemental Table 1. List of primers used.

PrimerID	Sequence	Description
	**Genotyping Primers**	
JB8046	ACCCAACACCCGTGCGTTTTATT	Intron-flanking forward primer
JB8047	TATATCACCGCCGACAAGCAGAAGA	Intron-flanking reverse primer
JB8545	AAGACTACATTTCCCCCTCCCTATG	3' end of insertion forward primer
JB8547	TTAGCCAGAAGTCAGATGCTCAAGG	3' end of insertion reverse primer
JB8821	CAATGTGGTGAATGGTCAAATGGCG	GFP forward primer
JB8822	AGCGGGGCTTCGGTTGTACG	GFP reverse primer
WA440	CTTGCGTACTATTGTTTGTACAGATG	IR forward primer
WA441	CATCACTGTTGGCTCTCCTTTAGTT	IR reverse primer
	**Inverse PCR Primers**	
JB8822	AGCGGGGCTTCGGTTGTACG	Inverse PCR primer 1
JB8897	CTAGGTCGGATCCTTTTCCCTCTG	Inverse PCR primer 2
	**Ligation Mediated PCR**	
WA050	CGAAGAGTAACCGTTGCTAGGAGAGACCGTG GCTGAATGAGACTGGTGTCGACACTAGTGG	long adapter for all ligations
WA051	GATCCCACTAGTGTCGACACCAGTCTCTAATT TTTTTTTTCAAAAAAA	short adapter for Sau3A1
WA052	CGCCACTAGTGTCGACACCAGTCTCTAATTTT TTTTTTCAAAAAAA	short adapter for MspI
WA224	TACCACTAGTGTCGACACCAGTCTCTAATTTTT TTTTTCAAAAAAA	short adapter for CviQI and MseI
WA225	AATTCCCACTAGTGTCGACACCAGTCTCTAAT TTTTTTTTTCAAAAAAA	short adapter for EcoRI
WAsph	CATGCCACTAGTGTCGACACCAGTCTCTAATT TTTTTTTTCAAAAAAA	short adapter for SphI and NcoI
WA053	CGAAGAGTAACCGTTGCTAGGAGAGACC	"splink1"- primer in long adapter
WA054	GTGGCTGAATGAGACTGGTGTCGAC	"splink2"- nested primer in long adapter
WA198	Biotin-TEG-CTAGGTCGGATCCTTTTCCCTCTG	Biotin-labeled forward primer in L1 sequence
WA510	Biotin-TEG- TTCCCCCTCCCTATGCAAAAGC	Biotin-labeled reverse primer in SB sequence
WA511	CCGCGTACAACCGAAGCC	IR forward primer used in second round of SB splink
	**5' and 3' Junction Genotyping Primers**	
JB8939	CTCTGCCAAAAATTATGGGGACATC	Forward primer in L1 sequence
JB8940	CCTTGAGCATCTGACTTCTGGCTAAT	Forward primer in L1 sequence
WA308	GTTTGGGGTCATTTAAAGGTATAGG	Reverse primer in 3' gDNA of hopB1718/1712
WA239	TTTCTCAGAACAGCCCAAGG	Reverse primer in 3' gDNA of hopB1919
WA223	TTGTGTTTTAAGTGGTAAATAAATAATTTG	Reverse primer in 3' gDNA of hopB1498
WA525	GTCAGGTTCTGAGGGTTGGA	Jump32 3’ end reverse primer
WA542	GTTACAAGTCTGGATTCCTTCCTG	Jump33 3’ end reverse primer
	**Bisulfite Sequencing Primers**	
WA083	ATGGGGGTGTTTTGTTGGTA	GFP exon 1 forward (anneals to exon 2)
WA197	AACCATTCACCACATTACTAACAAC	GFP exon 1 reverse (anneals to RSV LTR)
WA245	AGTTTTATAGGGTAAGTAGGTAAAAA	hopB1498 – Empty forward
WA248	AAAATAAAAAATTTATTTTTATACCTCTAT	hopB1498 – Empty reverse
WA245	AGTTTTATAGGGTAAGTAGGTAAAAA	hopB1498 – 5' Filled forward
WA261	ATTTTATTCCCACTTCTAAAAAAAA	hopB1498 – 5' Filled reverse
WA110	ATTATATTTTGAGGGGATTTTTAAT	hopB1498 – 3' Filled forward
WA248	AAAATAAAAAATTTATTTTTATACCTCTAT	hopB1498 – 3' Filled forward
WA189	AGGTGTGAATTATTATATTTAGTTAA	hopB1718 – Empty forward
WA190	AAAAAAACATTACTCTCCCCAATCT	hopB1718 – Empty reverse
WA110	ATTATATTTTGAGGGGATTTTTAAT	hopB1718 – 3' Filled forward
WA190	AAAAAAACATTACTCTCCCCAATCT	hopB1718 – 3' Filled reverse
WA237	TTGATGATGAATTTAGTAGGGGTAGA	hopB1919 – Empty forward short amplicon
WA236	AAACCAATACATTCCCAAAATACAA	hopB1919 – Empty reverse short amplicon
WA237	TTGATGATGAATTTAGTAGGGGTAGA	hopB1919 – Empty forward long amplicon

WA235	CCCTAACCTCAACTAACTTATACCC	hopB1919 – Empty reverse long amplicon
WA110	ATTATATTTTGAGGGGATTTTTAAT	hopB1919 – 3’ Filled forward
WA235	CCCTAACCTCAACTAACTTATACCC	hopB1919 – 3’ Filled reverse
WA399	AGTTGTAGGTGTTGAAGGAGAAGTAG	hopB1919 – ORF1 forward
WA400	CCCTTATAAATCACCTAACCCTTCT	hopB1919 – ORF1 reverse
WA385	TTAAGATTATTTAGTGGAAGAAGGATAGTA	hopB1919 – ORF2 forward
WA386	CCCACCAACAATACAACAAAATAC	hopB1919 – ORF2 reverse
WA387	TGGTTGGTTATAAGATTAATAGTAATAAGA	hopB1919 – ORF2 forward
WA388	AACCAATTATACCAACACCAATTAT	hopB1919 – ORF2 reverse
WA389	GGTATGTAGGGTTGGTTTAATATT	hopB1919 – ORF2 forward
WA390	AATCCTCCTTAATCTCCTTCTTCAA	hopB1919 – ORF2 reverse
WA391	TGTATAGTATTAAGTTGGAGAATTTGGA	hopB1919 – ORF2 forward
WA392	CCAAAACCTTAATCATAAAAAAATACTAAA	hopB1919 – ORF2 reverse
WA393	TTATTTGGAAGTTGAATAATATTTTGTTG	hopB1919 – ORF2 forward
WA394	TTCAACAAATCCACCTAATCCTAAT	hopB1919 – ORF2 reverse
WA397	AATAATTATTTTAGTTTGATTAGTTTGAAT	hopB1919 – ORF2 forward
WA398	ATCAAATCCATCTACTTCATCACCT	hopB1919 – ORF2 reverse

Supplemental Table 2. Overview of methylomes analyzed.

Sample	Species	Source of raw data	Source processed data	Accession number	Genome assembly
sperm	mouse	Kobayashi 2012	Kobayashi 2012	DRA000484	mm9
sperm	mouse	Wang 2014	Wang 2014	GSE56697	mm10
10.5 mPGC	mouse	Kobayashi 2013	Kobayashi 2013	DRA000607	mm9
13.5 mPGC	mouse	Kobayashi 2013	Kobayashi 2013	DRA000607	mm9
16.5 mPGC	mouse	Kobayashi 2013	Kobayashi 2013	DRA000607	mm9
2 cell	mouse	Wang 2014	Wang 2014	GSE56697	mm10
4 cell	mouse	Wang 2014	Wang 2014	GSE56697	mm10
ICM	mouse	Wang 2014	Wang 2014	GSE56697	mm10
E6.5	mouse	Wang 2014	Wang 2014	GSE56697	mm10
E7.5	mouse	Wang 2014	Wang 2014	GSE56697	mm10
liver	mouse	Hon 2013	Hon 2013	GSE42836	mm9
sperm	human	Molaro 2011	Ziller 2013	GSE46644	hg19
liver	human	http://www.roadmapepigenomics.org/data	Ziller 2013	GSE46644	hg19
colon	human	Ziller 2013	Ziller 2013	GSE46644	hg19
hippocampus	human	http://www.roadmapepigenomics.org/data	Ziller 2013	GSE46644	hg19
HUES64	human	Gifford 2013	Ziller 2013	GSE46644	hg19
ectoderm	human	Gifford 2013	Ziller 2013	GSE46644	hg19
endoderm	human	Gifford 2013	Ziller 2013	GSE46644	hg19
mesoderm	human	Gifford 2013	Ziller 2013	GSE46644	hg19
